# Different genome-wide transcriptome responses of *Nocardioides simplex* VKM Ac-2033D to phytosterol and cortisone 21-acetate

**DOI:** 10.1186/s12896-021-00668-9

**Published:** 2021-01-13

**Authors:** Victoria Yu Shtratnikova, Mikhail I. Sсhelkunov, Victoria V. Fokina, Eugeny Y. Bragin, Andrey A. Shutov, Marina V. Donova

**Affiliations:** 1 Belozersky Institute of Physico-Chemical Biology, Lomonosov Moscow State University, Leninskie gory, h. 1, b. 40, Moscow, Russian Federation 119991; 2 Skolkovo Institute of Science and Technology, Nobelya str., 3, Moscow, Russian Federation 121205; 3 Institute for Information Transmission Problems, Russian Academy of Sciences, Bolshoy Karetny per., h. 19, b. 1, Moscow, Russian Federation 127994; 4 G.K. Skryabin Institute of Biochemistry and Physiology of Microorganisms, Federal Research Center “Pushchino Center for Biological Research of the Russian Academy of Sciences”, pr. Nauki, 5, Pushchino, Moscow Region, Russian Federation 142290; 5 Pharmins, Ltd., R&D, Institutskaya str, 4, Pushchino, Moscow Region, Russian Federation 142290

**Keywords:** *Nocardioides simplex*, *Arthrobacter simplex*, *Pimelobacter simplex*, Biocatalysts, Transcriptome, Phytosterol, Cortisone acetate, Progesterone

## Abstract

**Background:**

Bacterial degradation/transformation of steroids is widely investigated to create biotechnologically relevant strains for industrial application. The strain of *Nocardioides simplex* VKM Ac-2033D is well known mainly for its superior 3-ketosteroid Δ^1^-dehydrogenase activity towards various 3-oxosteroids and other important reactions of sterol degradation. However, its biocatalytic capacities and the molecular fundamentals of its activity towards natural sterols and synthetic steroids were not fully understood. In this study, a comparative investigation of the genome-wide transcriptome profiling of the *N. simplex* VKM Ac-2033D grown on phytosterol, or in the presence of cortisone 21-acetate was performed with RNA-seq.

**Results:**

Although the gene patterns induced by phytosterol generally resemble the gene sets involved in phytosterol degradation pathways in mycolic acid rich actinobacteria such as *Mycolicibacterium, Mycobacterium* and *Rhodococcus* species, the differences in gene organization and previously unreported genes with high expression level were revealed. Transcription of the genes related to KstR- and KstR2-regulons was mainly enhanced in response to phytosterol, and the role in steroid catabolism is predicted for some dozens of the genes in *N. simplex*. New transcription factors binding motifs and new candidate transcription regulators of steroid catabolism were predicted in *N. simplex*.

Unlike phytosterol, cortisone 21-acetate does not provide induction of the genes with predicted KstR and KstR2 sites. Superior 3-ketosteroid-Δ^1^-dehydrogenase activity of *N. simplex* VKM Ac-2033D is due to the *kstDs* redundancy in the genome, with the highest expression level of the gene *KR76_27125* orthologous to *kstD2,* in response to cortisone 21-acetate. The substrate spectrum of *N. simplex* 3-ketosteroid-Δ^1^-dehydrogenase was expanded in this study with progesterone and its 17α-hydroxylated and 11α,17α-dihydroxylated derivatives, that effectively were 1(2)-dehydrogenated in vivo by the whole cells of the *N. simplex* VKM Ac-2033D.

**Conclusion:**

The results contribute to the knowledge of biocatalytic features and diversity of steroid modification capabilities of actinobacteria, defining targets for further bioengineering manipulations with the purpose of expansion of their biotechnological applications.

**Supplementary Information:**

The online version contains supplementary material available at 10.1186/s12896-021-00668-9.

## Key points

- Role in phytosterol catabolism is predicted for some dozens of *N. simplex* genes
- Four *kstD* genes are induced that provide superior 1(2)-dehydrogenase activity of the strain
- Gene *kstD2 KR76_27125* is highly over-expressed in response to AcC
- Metabolism of cortisone acetate is not regulated by KstR-repressors
- New candidate transcription regulators of steroid catabolism were predicted

## Background

Steroids represent a huge class of specific organic molecules with many of them playing essential roles in all living systems. Diverse bacteria from different ecological niches have been evolved to degrade steroids as sources of carbon and energy, or detoxify exogenic steroids by their structural modifications. Among bacteria, actinobacteria excite a particular interest due to their comprehensive metabolic possibilities and high activity of the enzymatic systems towards various steroids.

Currently, degradation of sterols (such as cholesterol, or phytosterols) by actinobacteria is in the focus of intensive researches due to its exclusive role in pathogenicity of *Mycobacterium tuberculosis*, and established applications of the non-pathogenic species and their engineered derivatives in biotechnology for production of high-value steroids for the pharmaceutical industry.

**Fig. 1:**
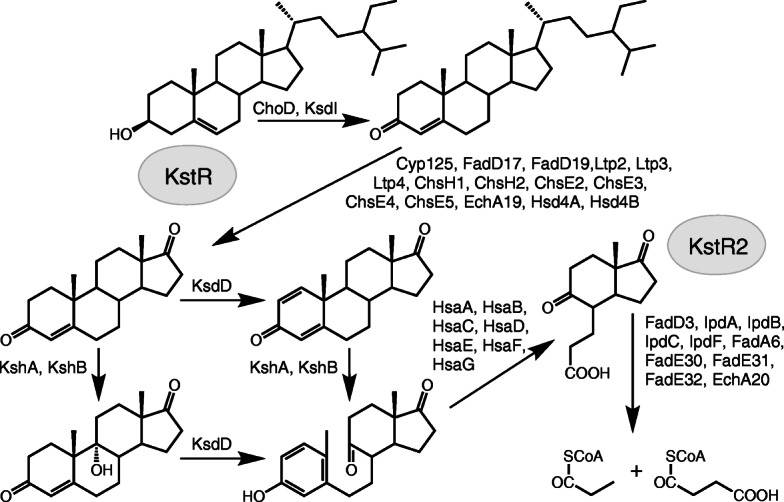
Putative scheme of phytosterol catabolism by *N. simplex* VKM Ac-2033D. “KstR” and “KstR2” in ovals mean regulons

Sterol catabolism is a complicated, multi-step process that included degradation of side chain, rings A/B and rings C/D of steroid core oxidation (Fig. 1). This pathway was reported to be controlled by two TetR-type transcriptional repressors, KstR and KstR2 [1–4].

Cholesterol catabolism has been intensively studied in the so-called mycolic acid rich actinobacteria such as pathogenic species: *Mycobacterium tuberculosis* [5], *Rhodococcus* strains [6, 7], as well as in the non-pathogenic species such as *Mycolicibacterium smegmatis* (basonym *Mycobacterium smegmatis*) mc^2^155 [4], *Gordonia cholesterolivorans* [8]. The roles of certain enzymes, mechanisms of their functioning and interactions with substrates were investigated. Cholesterol oxidases (ChOs) and/or 3β-hydroxysteroid dehydrogenases (3-HsDs) were shown to be responsible for modification of 3β-hydroxy-5-ene to 3-keto-4-ene- moiety that is considered as a first reaction of sterol degradation [4, 9]. Cytochrome P450 monooxygenases encoded by *cyp125, cyp124* and *cyp142* account for sterol hydroxylation at C-26(27) [10, 11], and aldol lyases encoded by *ltp3* and *ltp4* play role in the degradation of C24-branched chain sterols [12]. Acyl-CoA synthetase FadD19 catalyzes the formation of the cholestanoate CoA-thioester [13] that is further oxidized to C-17-ketosteroids by three successive cycles of β-oxidation [4, 14]. 9α-Hydroxylase (KshAB, consisting of KshA and KshB subunits) and 3-ketosteroid-∆^1^-dehydrogenase (KstD) [15, 16] are well-known as key enzymes of steroid core degradation whose cooperative action provides the ring B opening (Fig. 1). 3-Hydroxy-9,10-seconandrost-1,3,5(10)-triene-9,17-dione (3-HSA) formed is further degraded by the enzymes encoded by *hsaA*, *hsaB*, *hsaC*, *hsaD*, *hsaE*, *hsaF*, *hsaG* [17–19]. The degradation of the last two rings (rings C/D) of the steroid core is catalyzed by the products of the genes of the so-called KstR2-regulon [20]. Unlike mycobacteria, rhodococci and other above-mentioned species related to *Corynebacteriales* order, much less is known on steroid catabolism in actinobacteria belonging to *Propionibacteriales* order such as representatives of *Nocardioides* genus and related genera. The capability of *Arthrobacter simplex* (syn*. Nocardioides simplex*) to degrade cholesterol has been reported more than 50 years ago and the major intermediate was identified as cholest-4-ene-3-one [21]. Since then mostly 3-ketosteroid-Δ^1^-dehydrogenases (KstDs) from *A. simplex* were studied, the gene coding for these enzymes were identified and the engineered strains were constructed to provide selective production of 1(2)-dehydrosteroids (e.g. [22, 23]).

Recent metagenome research revealed among others the presence of the potent steroid degraders belonging to *Nocardiodaceae* and *Nocardioides* in different habitats [24]. Dozens of genes associated with steroid catabolism were detected in *Nocardioides dokdonensis* FR1436 isolated from sand sediment [25]. Most the research on the molecular mechanisms of steroid catabolism have been provided by our team for the biotechnologically relevant strain of *Nocardioides simplex* VKM Ac-2033D [26–28]. The data obtained in [26] have been further used for the refolding in situ of a soluble cholesterol oxidase PsChO3 *KR76_06800* (cholesterol oxidase ChoD) producing cholest-5-en-3-one [29], and for the rational design and protein engineering of cholesterol oxidase (PsChO) *KR76_21605* producing 3-oxo-4-ene-steroid products thus allowing significant improvement of the enzyme substrate selectivity [30].

Superior 3-ketosteroid-Δ^1^-dehydrogenase (KstD) activity of *Nocardioides simplex* VKM Ac-2033D towards various 3-ketosteroids provides effective production of the pharmaceutical 1(2)-dehydroanalogs [31–34]. It is known that the 1(2)-dehydroanalogs often demonstrate higher therapeutic efficiency and lesser side effects as compared with the corresponding 1(2)-saturated steroid drugs [35, 36]. The *N. simplex* VKM Ac-2033D is also capable of hydrolyzing steroid esters such as 21-acetylated steroids, reduction of C17 and C20 carbonyl groups of androstanes and pregnanes, respectively [31–33] and able to grow on cholesterol [27].

High KstD activity of *N. simplex* VKM Ac-2033D had been earlier demonstrated towards cortisol [37] and its 6-methylated derivative [38], androst-4-ene-3,17-dione (AD) and its 6-aminomethylated derivatives [34], testosterone [34], androsta-4,9-diene-3,17-dione [39], 21-acetoxy-pregna-4(5),9(11),16(17)-triene-21-ol-3,20-dione and pregna-4,9(11)-diene-17α,21-diol-3,20-dione acetates [32, 33], 7α- and 7β-hydroxytestololactone [40], etc. (more information is given in the Supplementary Table S1). In this study, the substrate spectrum of steroids for bioconversion with *N. simplex* was expanded with progesterone (Pr) and its 17α-hydroxylated and 11α,17α-dihydroxylated derivatives (17α-OH-Pr and 11α,17α-di-OH-Pr).

The strain had originally been isolated from the Pamir highland soils [41] and identified firstly as *Mycobacterium* sp. 193 [42], then re-classified as *Mycobacterium globiforme* 193 [43], and later as *Arthrobacter globiformis* 193 based on its cultural and morphological properties [44].

In 1976, Prauser had proposed a new genus *Nocardioides* for the Gram-positive, aerobic, non-sporeforming species, with *LL*-2,6-diaminopimelic acid in their cell walls and lack of mycolic acids [45]. Accordingly, the coryneform bacteria differed from other *Arthrobacter* species in their cell wall composition such as previously reported *Arthrobacter simplex* had been re-classified as belonging to *Nocardioides* genus [46]. Currently, more than 90 validly published species are within this genus [47]. The strain *Arthrobacter globiformis* 193 had been re-classified as *Nocardioides simplex* VKM Ac-2033D in 2003 [31].

Intriguingly, Suzuki and Komagata proposed the genus *Pimelobacter* (pimele oil - inhabiting; bacterium - a rod) for the *Arthrobacter* species with *LL*-2,6-diaminopimelic acid instead of lysine in the peptidoglycan layer with a type strain *Pimelobacter simplex* (syn. *Arthrobacter simplex*) [48]. As a result of taxonomic re-evaluation, *Pimelobacter simplex* was later re-classified as *N. simplex* [49, 50].

This complicated story resulted in the confusing situation when *Arthrobacter simplex*, *Pimelobacter simplex* and *Nocardioides simplex* names are often used as synonyms in the current literature for similar actinobacteria. In our case, a complete genome sequence of *N. simplex* VKM Ac-2033D was presented to NCBI database, and indicated by NCBI database as *Pimelobacter simplex* VKM Ac-2033D [26] under accession no. CP009896, its detailed bioinformatics analysis was carried out in [27].

In our previous study, the putative genes and gene clusters related to the sterol uptake system, aliphatic side chain degradation, A/B- and C/D-ring opening and degradation systems were revealed in the genome of *N. simplex* VKM Ac-2033D [27]. Previously unknown steroid metabolism gene clusters have been predicted. Orthologous clusters were later discovered also in *N. dokdonensis* FR1436^T^ [25].

**Fig. 2:**
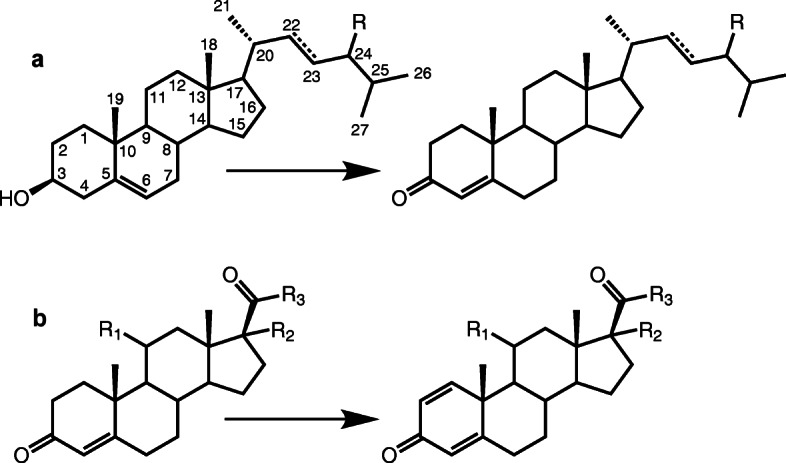
Major reactions of steroid bioconversion by *N. simplex*: **a**. Oxidation of 3-hydroxy group of phytosterol constituents: R = C_2_H_5_ β-sitosterol/stigmast-4-en-3-one (β-sitostenone); R = C_2_H_5_, Δ^22,23^stigmasterol/stigmasta-4,22-dien-3-one; R = CH_3_ campesterol/campest-4-en-3-one, R = CH_3_, brassicasterol/brassicast-4-en-3-one. **b**. R_1_ = H_,_ R_2_ = H_,_ R_3_ = CH_3_, Progesterone (Pr)/1(2)-dehydro-progesterone (DPr); R_1_ = H_,_ R_2_ = OH_,_ R_3_ = CH_3_, 17α-hydroxyprogesterone (17α-OH-Pr)/1(2)-dehydro-17α-hydroxyprogesterone (17α-OH-DPr); R_1_ = OH_,_ R_2_ = OH_,_ R_3_ = CH_3_, 11α,17α-dihydroxyprogesterone (11α,17α-di-OH-Pr)/1(2)-dehydro-11α,17α-dihydroxyprogesterone (11α,17α-di-OH-DPr); R_1_ = O_,_ R_2_ = OH_,_ R_3_ = CH_2_-O-C(O)-CH_3_ 21-acetate of cortisone or cortisone acetate (AcC)/21-acetate of 1(2)-dehydro-cortisone or 21-acetate of prednisone (AcP)

It is known that most bacterial enzyme systems involved in steroid metabolic pathways are mainly induced with different steroids, but not expressed constitutively [51]. In this study phytosterol and cortisone 21-acetate (AcC) were estimated as inducers. Phytosterol is a mixture of plant sterols with a branched side chain (with β-sitosterol as a main component) (Fig. 2). Unlike natural sterols such as cholesterol, or phytosterol, AcC is a synthetic corticosteroid ester which is often applied for induction of KstD activity in *N. simplex* and related strains [38, 52]. However, neither growth on AcC, no AcC bioconversion with *N. simplex* VKM Ac-2033D has been examined so far. The fundamental questions also are: whether AcC can be metabolized by *N. simplex* and whether the gene network that over-expressed in response to AcC corresponds to that of steroid degradation. To address these issues, we compared differential transcriptomes of *N. simplex* cultured with (and without) phytosterol, and with (and without) AcC.

Differentially expressed genes (DEG) sets in different induction conditions were compared and expression of the predicted steroid catabolic genes and their clusters was evaluated. The operons with highest expression level and new candidate transcription regulators motifs of steroid catabolism were predicted. The *kstD* gene with superior expression in response to AcC was revealed, and steroid conversion assay confirmed high level of KstD activity towards progesterone (a predicted preferable substrate for the product of gene *kstD2*) and progesterone derivatives. Moreover, the over-expressed genes encoding the enzymes related to the basic catalytic properties of the *N. simplex* VKM Ac-2033D were revealed.

## Results

### Steroid substrates and major bioconversion products

Structures of the steroid substrates used in this work and the bioconversion products detected are presented in Fig. 2. The corresponding 1(2)-dehydroderivatives were identified as major bioconversion products from Pr, 17α-OH-Pr and 11α,17α-di-OH-Pr and AcC. The main intermediates of phytosterol (comprising a mixture of β-sitosterol, stigmasterol, campesterol, brassicasterol) transformation by *N. simplex* cells were identified as corresponding phytostenones (stigmast-4-en-3-one (β-sitostenone), stigmasta-4,22-dien-3-one, campest-4-en-3-one, brassicast-4-en-3-one) (Supplementary Table S2 and S3). Earlier, campest-4-en-3-one, stigmast-4-en-3-one, and stigmasta-4,22-dien-3-one had been reported as intermediates at bioconversion of campesterol, β-sitosterol, stigmasterol, respectively, with *Arthrobacter simplex* IAM 1660 [21].

### Growth and steroid bioconversion

**Fig. 3:**
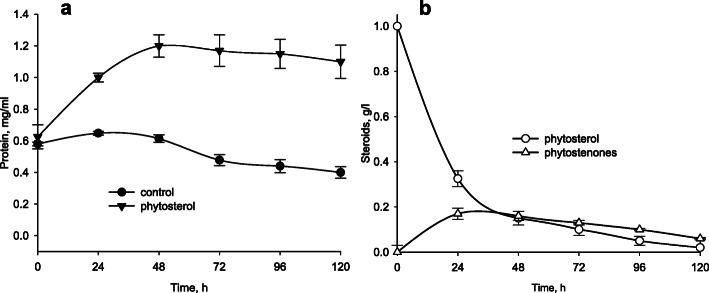
**a**: Growth of *N. simplex* VKM Ac-2033D on phytosterol. Medium without phytosterol was used in control. **b**: Phytosterol consumption and phytostenones accumulation during strain growth on phytosterol

As shown in Fig. 3a, *N. simplex* VKM Ac-2033D can grow on phytosterol while no growth was observed in the mineral medium with only MCD (control). As shown in Fig. 3b, phytosterol was completely degraded by *N. simplex* in approx. 120 h via transit intermediates (phytostenones).

**Fig. 4:**
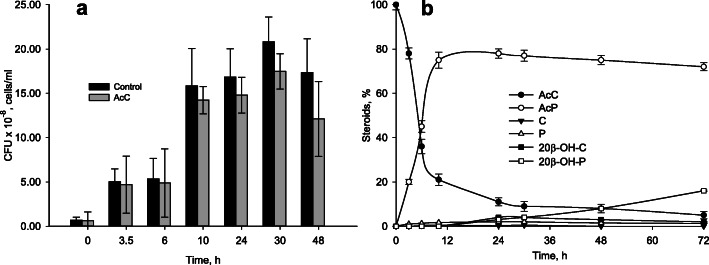
**a**: Growth of *N. simplex* VKM Ac-2033D in the presence of AcC. Media without AcC was used as a blank. **b**: Dynamics of AcC bioconversion. Data are the average of three replicates. Abbreviations: AcC – cortisone 21-acetate; C - cortisone, 20β-OH-C - 20β-hydroxy cortisone, 20β-OH-P - 20β-hydroxy prednisone

No growth difference was observed when *N. simplex* incubating with AcC in comparison with control (without AcC) (Fig. 4a). Maximum CFU number for AcC which was observed on 24 h was smaller than CFU number in the control: 17.4 × 10^8^ ± 2 × 10^8^ and 20.8 × 10^8^ ± 2.8 × 10^8^ cells/ml, respectively. Unlike phytosterols, AcC was not degraded by *N. simplex*, but converted into the mixture of steroids (21-acetate of prednisone, AcP (main product), prednisone (P), cortisone (C) and their 20-hydroxyderivatives (Fig. 4b)). Quantification of all steroids formed from AcC evidenced the absence of steroid core destruction. Expectedly, no AcP, or any other steroids were detected if no AcC was added (control) (data not shown).

### High-throughput mRNA sequencing

Complete transcriptomes of the *N. simplex* cells grown in the presence, or absence of phytosterol, as well as in the presence, or absence of AcC were obtained. The total number of reads, the percentage of reads mapping to the rRNA genes, and links to SRA repository in NCBI for experimental variants are provided in the Table 1.

**Table 1:** Sequencing statistics and links to reads

*Samples*	*Total number of reads*	*Percent of reads mapping to the rRNA genes*	*Link to the reads in SRA*
Phytosterol, 1st replicate	10,718,187	2	https://www.ncbi.nlm.nih.gov/sra/SRX5542009
Phytosterol, 2nd replicate	10,138,962	2.9	https://www.ncbi.nlm.nih.gov/sra/SRX5542008
AcC, 1st replicate	8,938,451	3	https://www.ncbi.nlm.nih.gov/sra/SRX5542005
AcC, 2nd replicate	11,883,597	4.4	https://www.ncbi.nlm.nih.gov/sra/SRX5542004
Control, 1st replicate	10,207,198	2.1	https://www.ncbi.nlm.nih.gov/sra/SRX5542007
Control, 2nd replicate	12,237,275	5	https://www.ncbi.nlm.nih.gov/sra/SRX5542006

### Differentially expressed genes (DEG)

DEG analysis showed 91 up- and 42 down-regulated genes in phytosterol plus conditions, while 81 and 31 genes were up- and down regulated in response to AcC, respectively (Table 2). A gene was considered differentially expressed, if its expression increased or decreased more than threefold with *q*-value less or equal to 0.01.

**Table 2:** Differentially expressed genes in gene clusters of *N. simplex* VKM Ac-2033D

Cluster	Genes	Phytosterol				AcC			
		I	Ave	Max	D	I	Ave	Max	D
А	*KR76_14160*-*KR76_14505*	31	10.94	31.24	0	0			0
B	*KR76_12190*-*KR76_12345*	12	6.90	14.70	0	0			0
C	*KR76_25015*- *KR76_25200*	14	15.10	59.00	0	0			0
D	*KR76_27035*-*KR76_27130*	7	13.35	24.53	0	3	415.5	1207.7	0
E	*KR76_17995*-*KR76_18070*	0	0.00		0	0			0
Out of clusters		27	7.06	12.44	42	79			32
Total		91	9.94		42	82			32

I - the number of genes that significantly (more than 3 times with *q*-value < 0.01) increased their expression

Ave - the average level of induction among the genes that significantly increased expression in the cluster

Max - the maximum level of induction

D - the number of genes that significantly (more than 3 times with q-value < 0.01) reduced their expression

In phytosterol induced cells, maximum expression level (59-fold) was observed for gene *KR76_25195* coding for ABC amino acid transporter; while the minimum level was indicated for *KR76_00525*, a putative two-component system sensor kinase, at 0.06 of phytosterol minus conditions. When inducing with AcC, maximum over-expression (1207-fold) was determined for the gene *KR76_27125* encoded KstD; and the minimum level was shown by *KR76_15900* RidA/YjgF/TdcF/RutC subgroup, at 0.03 of phytosterol minus conditions.

**Fig. 5:**
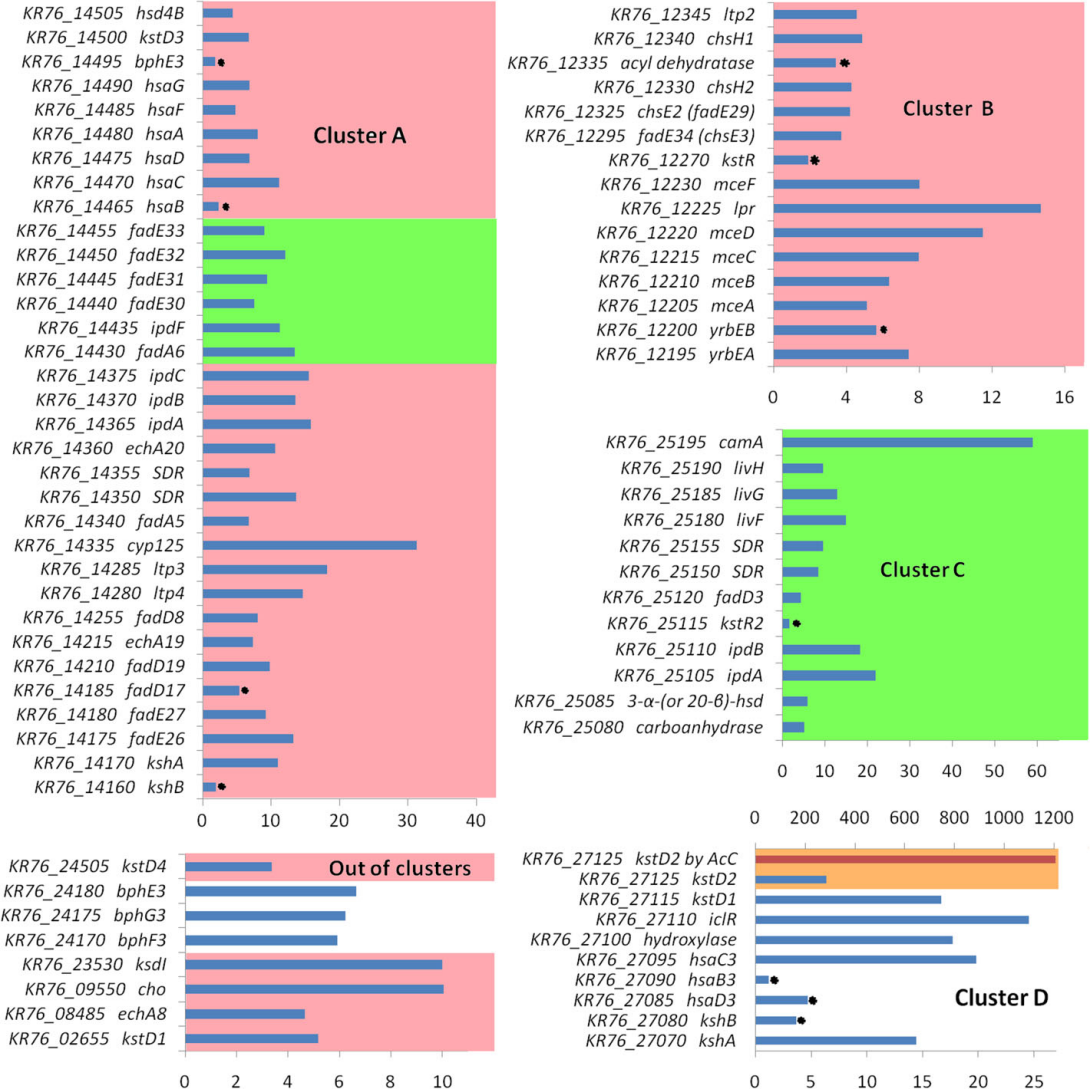
Induction of sterol catabolism genes by phytosterol and AcC in *N. simplex* VKM Ac-2033D. Blue bars – induction by phytosterols, red bar – induction by AcC. Pink color – predicted KstR regulon; green color – predicted KstR2 regulon; orange color – predicted Acin sites. The asterisks mark the changes from *q*-value over the threshold 0.01; the data for these genes are presented because they are related to steroid catabolism

The steroid induced genes were combined into the operons using bioinformatics methods, supported by the data from the transcriptome analysis. The detailed information about these operons, predicted binding motifs of transcription factors and levels of gene expression is provided in Supplementary Table S4. Induced genes with homology to the known genes of steroid catabolism are depicted in Fig. 5.

Almost two-thirds of the genes that were induced by phytosterol locate in the “typical” steroid metabolism clusters A and B (Table 2), in the newly cluster C that was previously predicted in [27], as well as in the cluster D, which does not have predicted binding motifs for the transcription factors KstR and KstR2.

The only gene that was highly up-regulated both in the presence of phytosterol and AcC, was *KR76_27125*, *kstD2* coding for KstD. The gene belongs to cluster D. Three genes with unknown functions were significantly down-regulated in phytosterol plus and AcC plus conditions (Supplementary Table S4). No cases of meaningful reverse gene behavior, when one inducer caused significant increase in expression, while another caused significant expression decrease, were detected.

Except for *kstD2,* no significant expression of the known genes putatively involved in the steroid degradation was observed in AcC plus conditions. No pronounced clustering of genes was observed either: the operons that enhanced expression were evenly distributed throughout the genome (Supplementary Table S4). A number of the AcC-induced genes could be combined into small clusters, specifically, *KR76_09390 - KR76_09460*; *KR76_16215 - KR76_16555*; *KR76_24345 - KR76_24405*; *KR76_24685 - KR76_24800*. These clusters included the genes related to various transporters, amino acid and organic acid metabolism enzymes, cytochromes, transcriptional regulators, dehydrogenases including 3-ketosteroid-Δ^1^-dehydrogenase etc.

### Expression of steroid catabolism genes orthologs and identification of candidates for new transcription factor regulators of steroid catabolism genes

Our previous study [27] allowed prediction of the KstR and KstR2 binding sites in 71 and 29 operons, respectively. Of these, in phytosterol plus conditions putative KstR-regulated genes of 27 operons (38%) and putative KstR2-regulated genes of 10 operons (34%) increased their expression. In response to AcC, no cases of gene induction with the predicted KstR or KstR2 binding site were observed.

Transcription of nine operons was enhanced in the presence of phytosterol, though the genes do not have predicted binding sites to KstR, or KstR2. Five of them (in which a total of seven genes increased expression significantly) located in cluster D (*KR76_27035-KR76_27130*).

To identify candidate motifs for the binding of transcription factors, we used the tool FIMO from the MEME 4.10.0 suite [53]. The search was carried out on the sections 500 bps upstream to 50 bps downstream. Identification was carried out for the operons that (i) increased, (ii) decreased their expression in AcC plus conditions and (iii) down-regulated in phytosterol plus conditions. Several motifs were identified (Supplementary Table S5): three motifs for the operons that increased expression on AcC (Acin1, Acin2 and Acin3) and one motif for the operons that decreased expression on phytosterol (Sitdec1). Nothing was found before the operons that reduced expression on AcC. The operons with these sites are listed in the Supplementary Table S4.

### Progesterone bioconversion

**Fig. 6:**
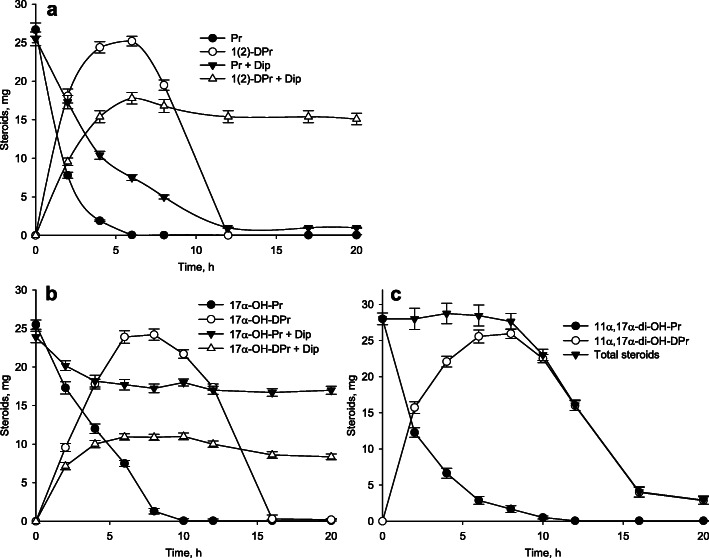
**a**: Bioconversion of progesterone (Pr, 0.4 g/l) with and without α,α-dipyridyl (Dip). **b**: Bioconversion of 17α-hydroxy-progesterone (17α-OH-Pr, 1 g/l) with and without α,α-dipyridyl. **c:** Bioconversion of 11α,17α-di-hydroxy-progesterone (11α,17α-di-OH-Pr, 1 g/l) without α,α-dipyridyl. In all variants resting AcC-induced *N. simplex* VKM Ac-2033D cells were used

As reported earlier, preferable substrate for KstD2 from *Rhodococcus ruber* is progesterone [54]. The *kstD2* from *N. simplex* has the moderate amino acid sequence similarity (69%) with that KstD2 from *R. ruber.* In this study, we tested KstD activity of *N. simplex* in vivo towards progesterone (Pr), and its hydroxylated derivatives, such as 17α-OH-Pr and 11α,17α-di-OH-Pr. As shown in Fig. 6, the corresponding 1(2)-dehydroderivatives were formed as major products from all three substrates. Their accumulation reached maximum level at 10 h, followed by the consequent degradation. Lower degradation was observed in the presence of α,α-dipyridyl (the inhibitor of iron containing enzymes).

## Discussion

### Sterol degradation

The genetics of sterol (phytosterol and β-sitosterol) degradation by mycolic-acid rich actinobacteria belonging to *Mycolicibacterium* (syn. *Mycobacterium*) (e.g. *M. smegmatis*, *M. neoaurum*), *Mycobacterium* (e.g. *M. tuberculosis*), *Rhodococcus*, *Gordonia* and other genera have been studied intensively during past two decades, while little information is known for the representatives of *Nocardioides* and related actinobacteria. Similar to many other soil-dwelling bacteria [55] *N. simplex* could be able to utilize abundant plant sterols such as sitosterol which is a core component of plant membranes. Indeed, *N. simplex* VKM Ac-2033D grows on phytosterol as a sole source of carbon and energy as it was confirmed in this study. Phytostenones were detected as major intermediates at phytosterol degradation by the *N. simplex* VKM Ac-2033D cells.

**Fig. 7:**
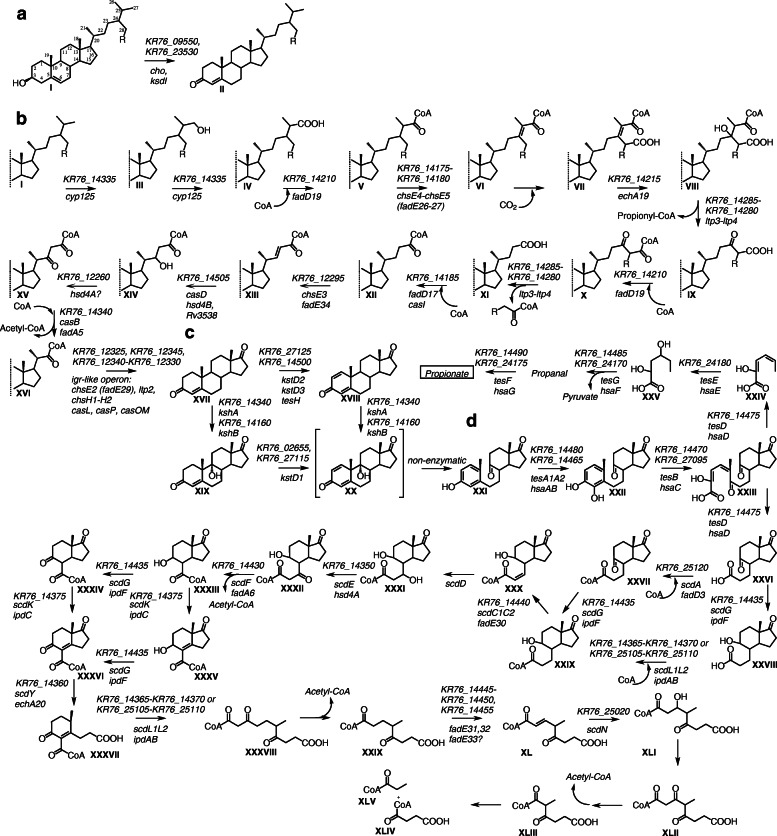
Biochemical scheme of sterol catabolism in *N. simplex* VKM Ac-2033D. Genes coding respective proteins are denoted, locus-tags from *N. simplex* VKM Ac-2033D are predicted, see Table 3. **a** Modification of 3β-ol-5-ene to 3-keto-4-ene moiety in A-ring of steroid core; **b** degradation of the C24-branched sterol side chain to C_19_-steroids; **c** steroid core modifications; **d** steroid core degradation via 9(10)-seco pathway. I - sterols; II - stenones. R = H, campesterol, campestenone; R = CH_3_, sitosterol, stigmast-4-en-3-one (β-sitostenone); XVII - androst-4-ene-3,17-dione (AD), XVIII *-* androsta-1,4-diene-3,17-dione (ADD), XIX - 9α-hydroxy-AD, XX - unstable 9α-hydroxy-ADD, XXI - 3β-hydroxy-9,10-*seco-*androsta-1,3,5(10)- triene-9,17-dione (3βHSA), XXII - 3,4-dihydroxy-9,10-*seco*androsta-1,3,5(10)-triene-9,17-dione (3,4-DHSA), XXIII - 4,5-9,10-di*seco*-3-hydroxy-5,9,17-trioxoandrosta-1(10),2-diene-4-oic acid (4,9-DSHA), XXIV - 2-hydroxyhexa-2,4-dienoic acid (2-HHD), XXV - 4-hydroxy-2-oxohexanoic acid, XXVI - 9,17-dioxo-1,2,3,4,10,19-hexanorandrostan-5-oic acid (DOHNAA) or 3a*α*-H-4α-(3′-propanoate)-7a*β*-methylhexahydro-1,5-indadione (HIP), XXVII - 9,17-dioxo-1,2,3,4,10,19-hexanorandrostan-5-oyl-CoA (HIP-CoA) XXVIII - 9-hydroxy-17-oxo-1,2,3,4,10,19-hexanorandrostan-5-oic acid or 3a*α*-*H*-4*α*(3′-propanoate)-5*α*-hydroxy-7a*β*-methylhexahydro-1-indanone (5-OH-HIP), XXIX - 9-hydroxy-17-oxo-1,2,3,4,10,19-hexanorandrostan-5-oyl-CoA (5-OH-HIP-CoA), XXX 9-hydroxy-17-oxo-1,2,3,4,10,19-hexanorandrost-6-ene-5-oyl-CoA (HIPE-CoA), XXXI - 7,9-dihydroxy-17-oxo-1,2,3,4,10,19-hexanorandrostan-5-oyl-CoA, XXXII - 9-hydroxy-7,17-dioxo-1,2,3,4,10,19-hexanorandrostan-5-oyl-CoA, XXXIII - 9-hydroxy-17-oxo-1,2,3,4,5,6,10,19-octa-*nor*androstan-7-oyl-CoA or 3a*α*-H-4*α*(carboxylCoA)-5*α*-hydroxy-7a*β*-methylhexahydro-1-indanone (5-OH-HIC-CoA), XXXIV - 9,17-dioxo-1,2,3,4,5,6,10,19-octa-*nor*androstan-7-oyl-CoA, XXXV - 9-hydroxy-17-oxo-1,2,3,4,5,6,10,19-octa-*nor*androst-8(14)-en-7-oyl-CoA, XXXVI - 9,17-dioxo-1,2,3,4,5,6,10,19-octa-*nor*androst-8(14)-en-7-oyl-CoA or 7a-Methyl-1,5-dioxo-2,3,5,6,7,7a-hexahydro-1H-indene-4-carboxylic acid (HIEC-CoA), XXXVII - 9-oxo-1,2,3,4,5,6,10,19-octanor-13,17-secoandrost-8(14)-ene-7,17-dioic acid-CoA-ester or (*R*)-2-(2-carboxyethyl)-3-methyl-6-oxocyclohex-1-ene-1-carboxyl-CoA (COCHEA-CoA), XXXVIII - 6-methyl-3,7-dioxo-decane-1,10-dioic acid-CoA ester, XXXIX - 4-methyl-5-oxo-octane-1,8-dioic acid-CoA ester, XL - 4-methyl-5-oxo-oct-2-ene-1,8-dioic acid-CoA ester (MOODA-CoA), XLI - 3-hydroxy-4-methyl-5-o xo-octane-1,8-dioic acid-CoA ester, XLII - 4-methyl-3,5-dioxo-octane-1,8-dioic acid-CoA ester, XLIII - 2-methyl-3-oxo-hexane-1,6-dioic acid-CoA ester, XLIV – succinyl-CoA, XLV – propionyl-CoA. Adopted from: [6, 12, 14, 17, 20, 60, 67, 75, 76, 79]

*N. simplex* is capable of full degradation of phytosterol and different other steroids without substituents in steroid core such as androstenedione, or progesterone (Supplementary Table S1). Genes whose products could play role in the reactions of steroid catabolism in *N. simplex* VKM Ac-2033D were predicted (Table 3, Fig. 7) based on their orthology with the known genes and new transcriptomic data.

**Table 3:** *N. simplex* VKM Ac-2033D genes whose products taking part in steroid degradation, predicted

Step	Enzyme annotation	*M. tub*	*R. jostii*	*C. testosteroni*	References	*N. simplex*	Cluster	Regulon	Phyt
*Regulation*									
	TetR transcriptional repressor	*kstR*			[2]	*KR76_12270*	B	KstR	
	TetR transcriptional repressor	*kstR2*			[2]	*KR76_25115*	C	KstR2	
*A-ring activation*									
I-II	cholesterol oxidase	*cho*			[56]	*KR76_09550*		KstR	*+*
I-II	∆^5^-∆^4^–3-ketosteroid isomerase	*ksi*			[57]	*KR76_23530*		KstR	*+*
					[57]	*KR76_24125*		KstR	
*Cholesterol side chain degradation*									
III-V	cytochrome P450 125	*cyp125*			[10, 58]	*KR76_14335*	A	KstR	*+*
V-VI	3-oxocholest-4-en-26-oate-CoA ligase	*fadD19*			[59]	*KR76_14210*	A	KstR	*+*
VI-VII	Acyl-coA dehydrogenases	*chsE4–5* (*fadE26–27*)			[60]	*KR76_14175-KR76_14180*	А	KstR	*+*
VII-VIII	2-enoyl-CoA hydratase	*echA19*			[6]	*KR76_14215*	A	KstR	*+*
IX-X; XI-XII	aldol lyase	*ltp3*			[12]	*KR76_14285*	А	KstR	*+*
	aldol lyase	*ltp4*				*KR76_14280*	А	KstR	*+*
XII-XIII	steroid-22-oyl-CoA synthetase	*fadD17*	*casI*		[61]	*KR76_14185*	A	KstR	
XIII-XIV	acyl-CoA dehydrogenase	*chsE3 (fadE34)*			[60, 62]	*KR76_12295*	B	KstR	*+*
XIV-XV	3α,7α,12α-trihydroxy-5β-chol-22-en-24-oyl-CoA hydratase	*hsd4B,* *Rv3538*	*casD*		[4, 61, 63]	*KR76_14505*	A	KstR	*+*
XV-XVI	dehydrogenase	*hsd4A*			[4]	*KR76_12260*	В	KstR	
XIV-XVII	3-ketoacyl-CoA thiolase	*fadA5*	*casB*		[64]	*KR76_14340*	А	KstR	*+*
XVII-XVIII; *igr-*operon	7α,12α-dihydroxy-23,24-bisnorchola-1,4-dien-22-oyl-CoA dehydrogenase	*chsE2 (fadE29)*	*casL*		[65]	*KR76_12325*	B	KstR	*+*
	acetyl-CoA acetyltransferase	*chsH2*	*casM*		[66, 67]	*KR76_12330*	B	KstR	*+*
	acyl dehydratase		*casN*		[66]	*KR76_12335*	B	KstR	
	beta-hydroxyacyl-ACP dehydratase	*chsH1*	*casO*		[66, 67]	*KR76_12340*	B	KstR	*+*
	3-ketoacyl-CoA thiolase	*ltp2*	*casP*		[66]	*KR76_12345*	B	KstR	*+*
*Opening of B-ring*									
XVIII-XIX; XX-XXI	Δ-3-ketosteroid dehydrogenase with preference for 9-OH-AD and testosterone	*kstD*	*kstD1*		[54, 68]	*KR76_02655*		KstR	*+*
						*KR76_27115*			*+*
	Δ-3-ketosteroid dehydrogenase with preference for progesterone	*kstD*	*kstD2*		[69]	*KR76_27125*	D	Acin1,3	*+*
	Δ-3-ketosteroid dehydrogenase with preference for saturated steroid substrates	*kstD*	*kstD3*	*tesH*	[54, 70]	*KR76_14500*	A	KstR	*+*
XVIII-XX; XIX-XXI	3-ketosteroid 9α-hydroxylase, oxygenase subunit	*kshA*			[71]	*KR76_14170*	A	KstR	*+*
						*KR76_27070*			*+*
	3-ketosteroid 9α-hydroxylase, reductase subunit	*kshB*				*KR76_14160*	A	KstR	
						*KR76_27080*			
*eCleavage of A-ring*									
XXII-XXIII	flavin-dependent monooxygenase	*hsaA*		*tesA1A2*	[18, 72]	*KR76_14480*	A	KstR	*+*
		*hsaB*				*KR76_14465*	A	KstR	
						*KR76_27090*	D		
XXIII-XXIV	extradiol dioxygenase	*hsaC*		*tesB*	[72, 73]	*KR76_14470*	A	KstR	*+*
						*KR76_27095*	D		*+*
XXIV-XXV	carbon–carbon hydrolase	*hsaD*		*tesD*	[19, 72]	*KR76_14475*	A	KstR	*+*
						*KR76_27085*	D		
XXV-XXVI	hydratase	*hsaE*		*tesE*	[17, 74]	*KR76_14495*	A	KstR	
						*KR76_24180*			*+*
XXVI-Propanal	4-hydroxy-2-oxovalerate aldolase	*hsaF*		*tesG*		*KR76_14485*	A	KstR	*+*
						*KR76_24170*			*+*
Propanal-Propionate	acetaldehyde dehydrogenase, acetylating	*hsaG*		*tesF*		*KR76_14490*	A	KstR	*+*
						*KR76_24175*			*+*
*Ring D degradation*									
XVII-XVIII	HIP-CoA synthetase	*fadD3*		*scdA*	[75, 76]	*KR76_25120*	С	KstR2	*+*
XVII-XIX	5-Oxo HIC-CoA oxidase	*ipdF*		*scdG*	[20, 76]	*KR76_14435*	A	KstR2	*+*
XXIX-XXX	COCHEA-CoA hydrolase, α-subunit	*ipdA*		*scdL1L2*	[20, 76]	*KR76_14365*	A	KstR2	*+*
						*KR76_25105*	С	KstR2	*+*
	COCHEA-CoA hydrolase, β-subunit	*ipdB*				*KR76_14370*	A	KstR2	*+*
						*KR76_25110*	С	KstR2	*+*
XXX-XXXI	acyl-CoA dehydrogenase	*fadE30*		*scdC1C2*	[76, 77]	*KR76_14440*	A	KstR2	*+*
XXXI-XXXII	acyl-CoA hydrolase			*scdD*	[76]				
XXXII-XXXIII	dehydrogenase, oxidized a hydroxyl in propionate chain	*hsd4A*		*scdE*	[76]	*KR76_14350*	A	KstR2	*+*
XXXIII-XXXIV	acetyl-CoA acetyltransferase	*fadA6*		*scdF*	[20, 76]	*KR76_14430*	A	KstR2	*+*
XXXIV-XXXV; XXXVI-XXXVII	5-Oxo HIC-CoA oxidase	*ipdF*		*scdG*	[20, 76]	*KR76_14435*	A	KstR2	*+*
XXXIV-XXXVI; XXXV-XXXVII	5-OH HIC-CoA reductase	*ipdC*		*scdK*	[20, 76]	*KR76_14375*	A	KstR2	*+*
*Ring C degradation*									
XXXVII-XXXVIII	HIEC-CoA hydrolase	*echA20*		*scdY*	[20, 76]	*KR76_14360*	A	KstR2	*+*
XXXVIII-XXXIX	CoA transferase	*ipdA*		*scdL1L2*	[20, 76]	*KR76_14365*	A	KstR2	*+*
						*KR76_25105*	С	KstR2	*+*
	CoA transferase	*ipdB*				*KR76_14370*	A	KstR2	*+*
						*KR76_25110*	С	KstR2	*+*
XL-XLI	acyl-CoA dehydrogenases	*fadE31, fadE32*	*ro04591-ro04593*		[61, 78]	*KR76_14445* *KR76_14450*	A	KstR2	*+*
	acyl-CoA dehydrogenases	*fadE33*				*KR76_14455*	A	KstR2	*+*
XLI-XLII	enoyl-CoA hydratase	*echA13*		*scdN*	[20, 76]	*KR76_25020*	С	KstR2	*+*

Step – degradation step from Fig. 7

Enzyme annotation – function of enzyme

*M. tub.* – name of gene in *M. tuberculosis* investigations

*R. jostii* - name of gene (or locus_tag) in *R. jostii* investigations

*C. testosteroni* - name of gene in *C. testosteroni* investigations

*N. simplex* - locus_tag of ortholog that is candidate gene for this function. Functions are predicted by the annotation, location, predicted regulon that ortholog belongs to and gene expression changes

Cluster – cluster where gene is located in genome [27]

Regulon – repressor whose binding site is predicted in the promoter of operon that ortholog belongs to

Phyt – gene in *N. simplex* VKM Ac-2033D that is induced by phytolesterol (+)

Bacterial steroid degradation pathway was shown to be controlled by two TetR-type transcriptional repressors named KstR and KstR2 [1–4]. In *Actinobacteria* 3-oxocholest-4-en-26-oic acid and its derivatives may regulate the transcription factor KstR [58], while the effector of KstR2 is HIP-CoA [80]. The reciprocal BLAST with the corresponding regulators in *M. tuberculosis* confirmed the amino acid sequence similarity of *KR76_12270* with KstR and *KR76_25115* with KstR2 [27].

As shown for saprophytic mycobacteria, side chain oxidation and ring B modification are the independent processes [81]. The alkyl side chain of sterols is cleaved according to the mechanism of fatty acid β-oxidation, which is triggered by hydroxylation at C26(27). Further oxidation of the side chain occurs with sequential cleavage of two- and three-carbon fragments. Steroid core degradation seems to carry out through the 9(10)-seco pathway with the B ring opening (see below). After that step sequential destruction of the rings A and B remnants, as well as the rings C and D occurs (Table 3, Fig. 7).

We considered in more details the genes related to the B ring opening. As mentioned above, the only known mechanism of bacterial steroid core degradation is the 9(10)-seco pathway which comprises 1(2)-dehydrogenation catalyzed by KstDs and 9α-hydroxylation by 3-ketosteroid-9α-hydroxylase (KshAB) (Fig. 7). The former enzymes encoded by *kstDs* [82], while the Rieske 3-ketosteroid-9α-hydroxylase consists of KshA monooxygenase and KshB reductase subunits encoding by *kshA* and *kshB*, respectively [71].

There are three *kshA* orthologs in the genome of *N. simplex* [27]. Transcription of two of them was enhanced in phytosterol induced cells, specifically, *KR76_14170* that located in cluster A (10.9-fold), and *KR76_27070* in cluster D (14-fold), which has no predicted *kstR* binding sites (Table 3). Noteworthy, none of them increased expression in response to AcC.

Broadest substrate range without clear substrate preference was reported for KshA5 of *Rhodococcus rhodochrous* DSM 43269 [83]. The enzyme showed activity towards different Δ^4^, Δ^1,4^, 5α-H and 5β-H steroids, steroids with bulky aliphatic side chains and was found to be an only KshA enzyme of the DSM 43269 strain that exhibited activity towards 5α-androstane-17β-ol-3-one and cortisol [83]. The closest ortholog of *kshA5* of *R. rhodochrous* DSM43269 is *KR76_27070* in *N. simplex* VKM Ac-2033D (identity 62%). Its product could be involved in progesterone (and other steroids) degradation by *N. simplex* via 9α-hydroxylation of their 1(2)-dehydrogenated intermediates. Unlike *R. rhodochrous* DSM 43269, the *N. simplex* VKM Ac-2033D does not degrade cortisol (Supplementary Table S1). It is possible that the product of *KR76_27070* is not active towards cortisol. The 3-keto-4-ene-steroid substrates substituted in positions 6, 7, 9, and 11 are considered more protected then unsubstituted 3-keto-4-ene-steroids from the action of the key enzymes involved in the degradation the steroid core; such ‘protected’ 3-keto-4-ene-steroids are widely used as the substrates in bioconversions with *N. simplex* [34, 37, 40].

The amino acid alignment and conserved amino acid residues [71] of expressed KshAs of *N. simplex* are shown in Supplementary Figure S1. N240 in the catalytic domain is replaced with D240. Similar replacement was earlier observed for the KshA of *Mycolicibacterium* sp. VKM Ac-1817D (syn. *Mycobacterium* sp. VKM Ac-1817D) [84]. Since *N. simplex* VKM Ac-2033D is not capable of degrading 11-functionalised steroids (e.g. cortisol, prednisolone, cortisone), this replacement might have altered the catalytic site in *N. simplex*.

It is well known that the number of oxygenase (KshA) and reductase (KshB) subunits of 9α-hydroxylase can differ [83]. For instance, five genes coding for KshA and two kshB genes were revealed in the genome of *Mycobacterium* sp. VKM Ac-1817D. Interestingly, when *N. simplex* VKM Ac-2033D growing on phytosterol, two *kshA*s and one *kshB* were up-regulated [84].

The domain structure of KshB of *N. simplex* is similar to KshB from *R. erythropolis* strain SQ1 [85] (Supplementary Fig. S2)*,* with two replacements in a NAD binding domain of *KR76_14160* that might indicate disturbed functionality of *KR76_14160* consistent with its lower induction level *(*1.9-fold) (Table 4, Fig. 5).

**Table 4:** Differentially expressed genes encoding KstDs and KshA/B

Locus_tag	Ortholog in Mtb	Localization	Product	*q*-value Phyt/C^a^	Phyt/C	*q*-value AcC/C^b^	AcC/C
*KR76_14170*	*kshA*	Cluster А	3-ketosteroid-9α-hydroxylase	2.24E-55	10.9	1	1.3
*KR76_27070*	*kshA*	Cluster D	3-ketosteroid-9α-hydroxylase	3.15E-59	14.4	1	1
*KR76_14160*	*kshB*	Cluster А	3-ketosteroid-9α-hydroxylase	0.7	1.9	1	1.2
*KR76_27080*	*kshB*	Cluster D	3-ketosteroid-9α-hydroxylase	1	3.7	1	1
*KR76_02655*	*kstD1*	Out of clusters	3-ketosteroid-Δ^1^-dehydrogenase	1.01E-07	5.2	1	1.4
*KR76_27115*	*kstD1*	Cluster D	3-ketosteroid-Δ^1^-dehydrogenase	1.4E-131	16.7	1	1.2
*KR76_27125*	*kstD2*	Cluster D	3-ketosteroid-Δ^1^-dehydrogenase	1.44E-14	6.4	0	1207.7
*KR76_01140*	*kstD3*	Out of clusters	3-ketosteroid-Δ^1^-dehydrogenase	1	0.3	1	0.3
*KR76_14500*	*kstD3*	Cluster А	3-ketosteroid-Δ^1^-dehydrogenase	1.12E-12	6.7	1	1

^a^Phyt/C – expression changes on Phytosterol with respect to control

^b^AcC/C – expression changes on AcC with respect to control

With regard to KstDs, five candidate *kstD* genes were earlier revealed in the genome of *N. simplex* that are divided into three orthogroups, specifically, *kstD1*, *kstD2* and *kstD3* [27].

KstD1 of *Rhodococcus erythropolis* PR4 and *R. ruber* shows substrate preference for 9-OH-AD and testosterone [54]. The orthologs of *kstD1* are *KR76_02655* and *KR76_27115* which increased their expression in phytosterol plus conditions.

KstD2 of *R. ruber* showed maximum preference for progesterone [54], while KstD2 of *Gordonia neofelifaecis* NRRL B-59395 transformed progesterone and cholest-4-en-3-one [69]. We tested KstD activity of *N. simplex* in vivo towards Pr, and its hydroxylated derivatives, such as 17α-OH-Pr and 11α,17α-di-OH-Pr. The corresponding 1(2)-dehydroderivatives were formed (Fig. 6). Lower degradation was observed in the presence of α,α-dipyridyl (the inhibitor of the iron containing enzymes, such as KshA). We suppose that progesterone bioconversion might be carried out by the *kstD2 KR76_27125* product. Amino acid sequence of KstD2 *KR76_27125* of *N. simplex* differ from consensus KstD2 [86] in some residues (Supplementary Fig. S3). Role of such substitution is not clear at the moment.

KstD3 of *R. ruber* preferred saturated steroid substrates (such as 5α-androstane-17β-ol-3-one) followed by progesterone [54]; KstD3 of *Gordonia neofelifaecis* showed higher activity to 16α,17α-epoxyprogesterone > AD > cholest-4-en-3-one > progesterone [69]. The expression of *kstD3* ortholog *KR76_14500* in *N. simplex* was increased on phytosterol 6.7-fold and was not increased in response to AcC.

Thus, effective 1(2)-dehydrogenation of 3-ketosteroids by *N. simplex* VKM Ac-2033D (Supplementary Table S1) is due to the presence of five *kstDs*, of which four genes are over-expressed in response to phytosterol and one (*kstD2*) is induced with AcC, but its transcription level is highest among all genes (more than 1200-fold).

Therefore, the genome-wide transcriptome analysis confirmed the transcriptional activation of the gene clusters related to the 9(10)-seco pathway of steroid catabolism in phytosterol induced *N. simplex* cells.

### Cortisone 21-acetate conversion

Unlike phytosterol, AcC could not be utilized by *N. simplex* VKM Ac-2033D as a carbon and energy source (Fig. 4a), but was converted mainly to its 1(2)-dehydroanalog - AcP (Fig. 4b). Probably, the presence of 11-oxo group in the molecule of AcC (or 11β-hydroxyl group in cortisol) hinders the action of 9α-hydroxylase (KshAB). These results are in accordance with the previously reported *N. simplex* activity towards cortisol and its derivatives [34, 37], and correlate with the data presented in Table 4 demonstrating quite different gene expression under phytosterol and AcC induction.

Along with the KstD activity, slight deacetylase and 20β-reductase activities were observed to form cortisone (C), prednisone (P), and 20β-reduced derivatives (Fig. 4b).

**Fig. 8:**
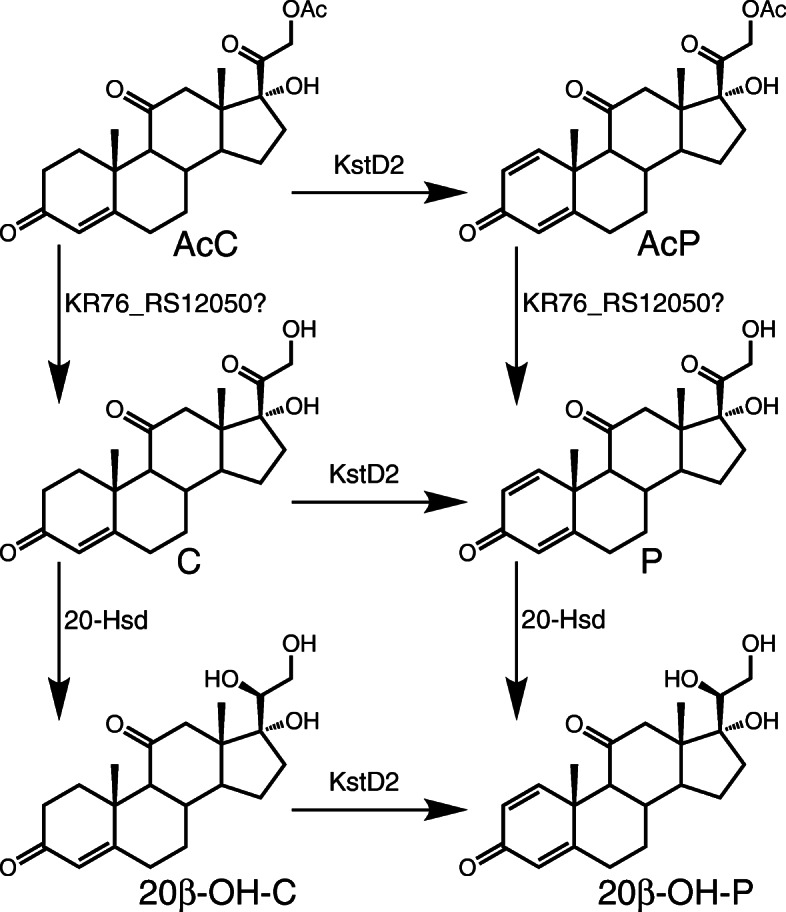
*N. simplex* VKM Ac-2033D AcC bioconversion scheme. Designations: AcP – 21-acetate of 1(2)-dehydro-cortisone or 21-acetate of prednisone; C – cortisone; P – prednisone; 20β-OH-C - 20β-hydroxy cortisone; 20β-OH-P - 20β-hydroxy prednisone

As shown earlier, efficiency of the 1(2)-dehydrogenation by *N. simplex* VKM Ac-2033D did not depend on the presence of acetyl group in the steroid inducer [31]. The order of deacetylation and 1(2)-dehydrogenation reactions can differ depending on the induction conditions (Fig. 8). In the AcC induced cells, 1(2)-dehydrogenation preceded deacetylation that coincides with the highest level of *kstD2* expression (Table 4), while in the absence of AcC induction, the reverse order of reactions was observed (data not shown).

Both cytosol-dissolved and membrane-associated constitutive steroid esterases were earlier reported for *N. simplex* [32]. Among the genes whose transcription is enhanced in response to AcC, no inducible esterases were detected. However, the analysis of the complete transcriptome revealed *KR76_12050* acyl-CoA thioesterase II with a high level of constitutive expression. Suggesting that the product of this gene might be responsible for 21-deacetylation we check a domain composition of the corresponding protein using InterProScan [87]. Since no membrane-bound domains were detected in this protein, the cytosolic enzymes are most likely responsible for the hydrolysis of 21-acetyl group in AcC.

Searching of the genes related to 20-reductase activity was carried out with protein BLAST. In *N. simplex* genome, three homologs of *fabG3* coding for 3α,20β-hydroxysteroid dehydrogenase [88] were revealed. Of these, expression of *KR76_13560* is greatly enhanced in response to AcC (13.7-fold) (Supplementary Table S4). Product of this gene is similar (amino acid sequence) by 56% with the cortisone-20β-reductase from *Streptomyces hydrogenans* [89].

FabG of *Bacillus megaterium* was identified as the enzyme with 20β-HSD activity [90] that reduce the β-ketoacyl group to a β-hydroxy group. Only one of homologs in *N. simplex* VKM Ac-2033D, namely, *KR76_14350*, increased its expression in the presence of phytosterol (Supplementary Table S4). None of these genes was induced by AcC.

Probably, the product of the gene *KR76_13560* may be responsible for the 20β-reducing activity of *N. simplex*. As shown earlier by Medentsev et al. [91], steroid 1(2)-dehydrogenation by *Arthrobacter grobiformis* (syn. *N. simplex*) did not depend on the level of NAD(P) H, but the resulting redox equivalents transferred to the respiratory chain to generate transmembrane potential (ΔμH^+^). Hypothetically, accumulated reduced equivalents may stimulate constitutive NADH-dependent 20β-HSD activity.

### Transcription factors binding motifs

Totally, about one third part of the operons was induced in which the predicted binding motifs for KstR and KstR2 were found earlier [27]. We have three possible explanations for such a low percentage. i) Some of the operons may be repressed by other transcription factors aside from KstR and KstR2. Even after KstR and KstR2 unbind, those other factors might continue to repress the operons. ii) Some of the predicted KstR and KstR2 binding sites could be false positive predictions. iii) If two operons are directed outward (“← →”), the intergenic region between them is small and a KstR or KstR2 site is present there, we supposed that it regulates both operons, while in fact it would regulate one of them. The relative contributions of these three effects were not estimated in this study.

Search for motifs for the binding of candidate factors revealed several recurring motifs before operons that strengthened, or weakened their expression.

Two sites (Acin1, Acin3) were found before *KR76_27125*, *kstD2*, with a maximum observed level of induction (Fig. 5). On the other side of these sites the transcriptional regulator gene *KR76_27130* belonging to the TetR family is located whose expression was induced 10-fold. The increased expression of this gene and the absence of the predicted binding sites for the transcription factors KstR and KstR2 in this area suggest that *KR76_27130* could be involved in the regulation of *kstD2* induction and that the predicted motifs belong to it. Recently, the TetR family transcriptional factor *KR76_27130* was confirmed to be a steroid catabolism regulator named SRTF1 (Steroid Responsive Transcriptional Factor 1) [92]. Its binding site coincided with the predicted Acin1 motif.

Other genes annotated as transcription factors that induced by AcC, specifically *KR76_27330* (transcriptional regulator, MerR family) and *KR76_26400* (transcriptional regulator, PadR family), both are located in an operon with predicted Acin3 site; and *KR76_24405* (putative transcriptional regulator) is in an operon with the predicted Acin1 site. However, the found Acin and Sitdec1 motifs are hardly enough to explain the change in the expression of all the operons upon the addition of phytosterol, or AcC. It seems that there would be another transcription factor with a small number of motifs that could not be detected by MEME tools. Post-transcriptional regulation of mRNA from these operons also cannot be fully excluded.

Nevertheless, the calculations and predictions indicate the most likely candidate genes that can serve as a target for future biotechnological research.

In the future, it is planned to expand the experiments by studying of a range of new inducers and increasing the number of replicates. New inducers will clarify the role of genes that are homologous to already known steroid catabolism genes but do not have binding motifs with its confirmed regulators. It is interesting to use as inducers substances that are intermediates at various stages of steroid catabolism, for example, androstenedione or androstendiendione. This will make it possible to determine the complexes of genes that are activated at later stages and to compare the catabolism of steroids in *N. simplex* VKM Ac-2033D with catabolism in more studied organisms. Knockout and proteomic experiments are also promising, confirming the role of calculated transcription factors.

## Conclusion

The gene network involved in phytosterol degradation by *N. simplex* VKM Ac-2033D is generally similar to those described for mycobacteria and related actinobacteria, but differs mainly by the existence of a cluster that has its own system of regulation.

In this study, we have generated experimental and computational evidences for the functionality of the clusters A, B, C, D those were identified by the bioinformatics analysis of the genome [27]. While the clusters A and B have been previously described for other bacteria, the clusters C and D were detected only by the analyzing of the *N. simplex* VKM Ac-2033D genome. Probably, the cluster D has its own previously unreported regulation system. We predicted a role in phytosterol catabolism for some dozens of genes of *N. simplex* VKM Ac-2033D; the genes taking part in AcC bioconversion were also suggested.

Our analysis suggests that the bioconversion of cortisone acetate by *N. simplex* is not regulated by the KstR-repressor.

The 3-ketosteroid-Δ^1^-dehydrogenase activity of *N. simplex* VKM Ac-2033D is firstly shown for the progesterone structure. The extremely high level of 1(2)-dehydrogenation of 3-ketosteroids is explained by the multiplicity and high level of induction of 3-ketosteroid-Δ^1^-dehydrogenases (*kstD2 KR76_27125* in the case of AcC). A number of genes that can be responsible for the increased 20β-reducing activity have been found, however, in the case of different inducers, transcription of different genes is enhanced.

The knowledge of the genome organization, the functionality of the existing sterol catabolism genes and their possible involvement in steroid oxidation process contributes to our understanding of steroid bioconversion by actinobacteria, as well as to the expansion of the application field of the *N. simplex* VKM Ac-2033D in biotechnology.

## Materials and methods

### Reagents

Phytosterol (total sterols content – 95.47%; β-sitosterol – 42.39%, stigmasterol – 26.08%, campesterol – 23.48%, brassicasterol – 3.52%) was obtained from Jiangsu Spring Fruit Biological Products Co., Ltd., China. Pregn-4-ene-3,20-dione (progesterone, Pr), pregn-4-en-17α-ol-3,20-dione (17α-hydroxy-progesterone, 17α-OH-Pr), pregn-4-ene-11α,17α-diol-3,20-dione (11α,17α-dihydroxy-progesterone, 11α,17α-di-OH-Pr) were purchased from Sigma (USA). 21-Acetoxy-pregn-4-ene-17α,21-diol-3,11,20-trione (21-acetate of cortisone, cortisone 21-acetate, AcC) was purchased from Steraloids (USA), yeast extract from Difco (USA), soya peptone from HiMedia (India), randomly methylated β-cyclodextrin (MCD) from Wacker Chemie (Germany). Other materials and solvents were of analytical grade and purchased from domestic commercial suppliers.

### Microorganism

A strain of *Nocardioides simplex* VKM Ac-2033D was obtained from the All-Russian Collection of Microorganisms (VKM). For RNA extraction, *N. simplex* VKM Ac-2033D was cultured as described in [27] on the mineral medium (control) supplemented with (g/l): yeast extract - 1.0, glycerol - 5.0, MCD - 3.46, and the same medium supplemented with phytosterol (0.5 g/L), or AcC (0.2 g/L).

Cultivation was carried out aerobically on a rotary shaker (200 rpm) at 30 °C for 16 h. For steroid bioconversion experiments, the cells were harvested by centrifuge (4 °C, 8000×*g,* 30 min) and washed with sterile saline as described in [31].

### Growth estimations

Growth on phytosterol, or AcC as a sole source of carbon was estimated as described in [27] on the mineral medium supplemeted with MCD 17 (g/L) (control), phytosterol (1 g/L), or AcC (1 g/L).

Because of low phytosterol and AcC solubilities, optical or dry weight measurements were inaccurate, and the biomass grown upon phytosterol plus or AcC plus conditions was assayed by protein content or CFU estimation, respectively. The protein concentration was determined by the Lowry method with preliminary alkaline hydrolysis of biomass by adding 0.9 ml of 0.5 N NaOH to a 0.1 ml sample. For colony-forming units (CFU) counting, some broth samples were serially diluted with saline under vigorous agitation and plated on the solid LB medium. The experiments were carried out in no less than three replicates.

### Bioconversion of steroids by the resting cells, isolation and identification of steroids

Progesterone, 17α-hydroxy-progesterone, and 11α,17α-dihydroxy-progesterone (0.4, 1.0, and 1.0 g/l, respectively) were tested as the substrates for bioconversion by resting whole cells of *N. simplex* (0.4, 0.2 and 0.2 g/l, respectively). Biomass was prepared as described above (in 2.2). Bioconversions were carried out in 0.02 М Na-phosphate buffer, рН 7.0, aerobically (200 rpm) at 30 °C in 750-ml shake flasks containing 100 ml buffered media with MCD (3.5–8.0 g/l). Concentration of α,α-dipyridyl when added was 0.4 g/l. The experiments were carried out in triplicates.

Steroid metabolites were isolated by preparative TLC [34]. Chromatographic purity of the compounds was monitored by TLC. For TLC, DC-Fertigfolien ALUGRAM® SIL G / UV_254_10 × 20 cm plates (Macherey-Nagel, Germany) were used. Steroids were developed in benzene:acetone 3:1 (v/v) or 4:1 (v/v) and visualized under UV-light (254 nm) in the chemiscope CN-6 (VilberLourmat, Germany).

HPLC analyses of steroid bioconversions were performed on a 5 μm C18 reverse phase column Symmetry 4.6 × 250 mm with a guard column Symmetry C18, 5 μm, 3.9 × 20 mm (Waters, Ireland). Mobile phase consisted of CH_3_CN:H_2_O:CH_3_COOH (40:60:0.01, v/v); at 50 °C, with a flow rate of 1 ml min^− 1^ and UV detection at 240 nm were used. Peaks were compared to authentic progesterone (P), 17α-hydroxy-progesterone (17α-OH-P), and 11α,17α-dihydroxy-progesterone (11α,17α-di-OH-P) (an external standard calibration method).

The structure of some steroids was confirmed by mass spectrometry (MS) and ^1^H-NMR spectroscopy. MS spectra of the reported steroids were recorded on a Finnigan LCQ Advantage MAX quadrupole ion trap mass spectrometer (Thermo Electron San. Jose, USA) in positive ions [M + H]^+^, at evaporator temperature 350 °C, capillar – 170 °C. MS/MS spectra were obtained using normalized collision energy (Normolized Collision EnergyTM) ranging from 20 to 40%. Collection and processing of data were performed using the Xcalibur software. Proton nuclear magnetic resonance (^1^H-NMR) spectra were recorded at 400 MHz with a Bruker Avance 400 spectrometer. Chemical shifts were measured relative to a solvent signal.

### Isolation of mRNA and high-throughput sequencing

For RNA isolation, the cells were taken in the exponential growth phase (16 h), harvested by centrifuge at 8000×*g* for 10 min and immediately ground in a porcelain mortar under liquid nitrogen. Total RNA was isolated using Qiagen RNeasy mini kit according to the instructions of the supplier (Qiagen, Netherlands). The resulting mixture was treated with DNAse I. The ribosomal RNA was depleted using Ribo-Zero rRNA Removal Kit (Bacteria) according to the protocols of the supplier (Epicentre, USA).

Sample preparation of mRNA for high-throughput sequencing was made with TruSeq RNA Sample prep kit v.2 according to the protocols of the manufacturer (Illumina, USA). Sequencing was conducted on HiSeq 2000 (50-nucleotide single-read run) according to the protocols of the manufacturer (Illumina, USA). The experiments were carried out in duplicate.

Real-time PCR validation was performed with using the AriaMx Realtime PCR system (Agilent, Richardson, TX, USA) with an M-439 kit (Eva Green I) (Syntol, Moscow, Russian Federation). The nucleotide sequences of primers used in this study for target and reference genes are listed in Supplementary Table S6. The amplification was performed as follows: 95 °C 5 min (1 cycle), 95 °C 10 s, 54 °C 15 s, 72 °C 30 s (40 cycles). Relative gene expression levels were calculated using the double delta Cq method [93].

### Computational analyses

The differential expression analysis was performed by Rockhopper 2.03 [94]. Rockhopper does not require the mapping of a whole read (mapping part of a read is enough). It was thus unnecessary to trim the reads before processing, and the raw reads were not trimmed. The reads were mapped by Rockhopper in the strand specific mode, with the seed length of 20 bp, allowing at most 1 mismatch between the mapped part of a read and the genome. The options “compute operons” (−y) and “identify transcript boundaries” (−t) were switched on. A gene was considered differentially expressed between the conditions phytosterol plus and phytosterol minus or AcC plus and AcC minus, if its expression increased or decreased more than threefold with *q*-value less or equal to 0.01.

To check the percentage of reads corresponding to rRNA, the reads were mapped to the rRNA genes of *N. simplex* by CLC Assembly Cell 4.2 (https://www.qiagenbioinformatics.com/products/clc-assembly-cell/); the reads were required to map with at least 98% sequence similarity.

The operon predictions made by Rockhopper were supplemented with the operon predictions made by FgenesB [95]. *M. tuberculosis* H37Rv was used as the “closest organism” in FgenesB. If Rockhopper and FgenesB provided different predictions for a particular operon, we chose the shortest prediction.

The predictions of KstR and KstR2 binding sites were taken from [27]. The search for membrane-associated domains in the protein corresponding to the gene with the locus tag *KR76_12050* was performed by the InterProScan [87] web server (https://www.ebi.ac.uk/interpro/interproscan.html) on August 5, 2018.

Alignments of the KshA, KshB and KstD proteins were done with CLC Genomics Workbench, v. 7.5.1.

Genes whose products could play role in the biochemical reactions were predicted based on the annotation, homology with the known enzymes (using orthogroups), gene expression changes, location and predicted regulons to which the orthologs belong.

Searching of the genes related to 20-reductase activity was carried out with protein BLAST for *fabG3* coding for 3α,20β-hydroxysteroid dehydrogenase [88] and *fabG* of *Bacillus megaterium* [90].

### Search of transcription factors binding motifs

To find out binding motifs of transcription factors that may be responsible for the change in operon expression, the following protocol was used:

1. Regions spanning from 500 bps upstream to 50 bps downstream of start codons of the first genes in the operons that changed their expression were taken.
2. In these regions, potential binding motifs were de novo predicted by MEME 4.10.0 [96]. The minimum allowed motif width was set to 8 bps, the maximum to 50 bps. Any number of motifs was allowed in each of the regions (the option “-mod anr”). Only motifs with *e*-value, less or equal to 0.1 were considered further.
3. Potentially, the found motifs may correspond to low complexity sequences, or promoters. To identify and discard such motifs, we took the regions spanning from 500 bps upstream to 50 bps downstream of start codons of all *N. simplex* genes and searched for the motifs corresponding to all the motifs found in the previous paragraph. The search was done by the tool FIMO from the MEME 4.10.0 suite [53] with the *q-*value threshold for reported motifs of 0.05. If a motif had 1000 sites or more, it was deemed unrelated to a transcription factor.
4. If a motif had less than 1000 sites, it was considered potentially corresponding to some transcription factor.

This protocol was applied separately three times: for the operons that (i) increased, or (ii) decreased their expression in AcC conditions, and (iii) decreased their expression in phytosterol conditions.

## Supplementary Information

**Additional file 1: Supplementary Table S1.** Steroid bioconversion by *N. simplex* VKM Ac-2033D. **Supplementary Table S2**.MS-characteristics of the steroid substrates and *N. simplex* bioconversion products. **Supplementary Table S3.** ^1^H-NMR spectra. **Supplementary Table S5.** New candidate motifs for the binding of transcription factors for steroid catabolism regulation in *N. simplex* VKM Ac-2033D. **Supplementary Table S6.** Real-time qPCR.

**Additional file 2: Supplementary Table S4.** DEGs and operons.

**Additional file 3: Supplementary Figure S1-S3.** Alignments of KshA, KshB, KstD [pdf]. **Supplementary Figure S1.** KshA alignment. **Supplementary Figure S2.** KshB alignment. **Supplementary Figure S3.** KstD alignment.

## Data Availability

The datasets supporting the conclusions of this article are included within the article and its additional files as Supplementary Table S3. Reads are in https://www.ncbi.nlm.nih.gov/sra/SRX5542009, https://www.ncbi.nlm.nih.gov/sra/SRX5542008, https://www.ncbi.nlm.nih.gov/sra/SRX5542005, https://www.ncbi.nlm.nih.gov/sra/SRX5542004, https://www.ncbi.nlm.nih.gov/sra/SRX5542007, https://www.ncbi.nlm.nih.gov/sra/SRX5542006.

## Abbreviations

MCD: methyl-β-cyclodextrin
AcC: cortisone 21-acetate
KstD: 3-ketosteroid-Δ^1^-dehydrogenase
DEG: differentially expressed gene(s)
