# Supplementary material for: Different genome-wide transcriptome responses of Nocardioides simplex VKM Ac-2033D to phytosterol and cortisone 21-acetate
Source: BMC Biotechnol. 2021 Jan 13;21:7. doi: 10.1186/s12896-021-00668-9 (PMC7807495; doi:10.1186/s12896-021-00668-9)
Supplement: Supplementary file 1 — Additional file 1: Supplementary Table S1. Steroid bioconversion by N. simplex VKM Ac-2033D. Supplementary Table S2.MS-characteristics of the steroid substrates and N. simplex bioconversion products. Supplementary Table S3. 1H-NMR spectra. Supplementary Table S5. New candidate motifs for the binding of transcription factors for steroid catabolism regulation in N. simplex VKM Ac-2033D. Supplementary Table S6. Real-time qPCR. [file 12896_2021_668_MOESM1_ESM.doc]

**Different genome-wide transcriptome responses of *Nocardioides simplex* VKM Ac-2033D to phytosterol and cortisone-21-acetate**

Victoria Yu. Shtratnikova^1*^, Mikhail I. Sсhelkunov^2,3^, Victoria V. Fokina^4,5^, Eugeny Y. Bragin^4^, Andrei A. Shutov^4,5^, Marina V. Donova^4,5^.

^1^Belozersky Institute of Physico-Chemical Biology, Lomonosov Moscow State University, Leninskie gory, h. 1, b. 40, Moscow, Russian Federation, 119991

^2^Skolkovo Institute of Science and Technology, Nobelya str., 3, Moscow, Russian Federation, 121205

^3^Institute for Information Transmission Problems, Russian Academy of Sciences, Bolshoy Karetny per., h. 19, b. 1, Moscow, Russian Federation, 127994

^4^G.K. Skryabin Institute of Biochemistry and Physiology of Microorganisms, Federal Research Center “Pushchino Center for Biological Research of the Russian Academy of Sciences”, pr. Nauki, 5, Pushchino, Moscow Region, Russian Federation, 142290

^5^Pharmins, Ltd., R&D, Institutskaya str., 4, Pushchino, Moscow Region, Russian Federation, 142290

**Supplementary Table S1** Steroid bioconversion by *N. simplex* VKM Ac-2033D.

| *Substrate* | *Bioconversion products* | *Steroid destruction* | *Reference* |
| --- | --- | --- | --- |
| Cholesterol | Cholestenone | + | [27] |
| Phytosterol  (β-Sitosterol, Stigmasterol, Campesterol, Brassicasterol) | Phytostenones  (Sitostenone, Stigmastenone, Campestenone, Brassicastenone) | + | [27]  This work |
| Androst-5-en-3β-ol-17-one (Dehydroepiandrosterone, DHEA) | Androst-4-ene-3,17-dione (AD)  Androsta-1,4-diene-3,17-dione (ADD)  Androst-4-en-17β-ol-3-one (Testosterone, T)  Androsta-1,4-dien-17β-ol-3-one (1(2)-Dehydro-testosterone, DT) | + | [97]* |
| 3β,7α-Dihydroxy-17a-oxa-*D*-homo-androst-5-en-17-one | 7α-Hydroxy-17a-oxa-*D*-homo-androst-4-ene-3,17-dione (7α-Hydroxy-testololactone)  7α-Hydroxy-17a-oxa-*D*-homo-androsta-1,4-diene-3,17-dione (7α-Hydroxy-testolactone) | + | [40] |
| 3β,7β-dihydroxy-17a-oxa-*D*-homo-androst-5-en-17-one | 7β-hydroxy-17a-oxa-*D*-homo-androst-4-ene-3,17-dione (7β-Hydroxy-testololactone)  7β-Hydroxy-17a-oxa-*D*-homo-androsta-1,4-diene-3,17-dione (7β-Hydroxy-testolactone) | + | [40] |
| 21-Acetoxy-pregn-4-ene-17α,21-diol-3,11,20-trione  (Cortisone 21-acetate, AcC) | 21-Acetoxy-pregna-1,4-diene-17α,21-diol-3,11,20-trione (21-Acetate of Prednisone)  Pregn-4-ene-17α,21-diol-3,11,20-trione (Cortisone)  Pregna-1,4-diene-17α,21-diol-3,11,20-trione (Prednisone)  Pregna-1,4-diene-17α,20β,21-triol-3,11-dione (20β-Reduced Prednisone) | - | This work |
| Pregn-4-ene-11β,17α,21-triol-3,20-dione  (Hydrocortisone) | Pregna-1,4-diene-11β,17α,21-triol-3,20-dione (Prednisolone)  Pregna-1,4-diene-11β,17α,20β,21-tetraol-3,20-dione (20β-Reduced Prednisolone) | - | [37] |
| 6α-Methyl-pregn-4-ene-11β,17α,21-triol-3,20-dione (6α-Methyl-hydrocortisone) | 6α-Methyl-pregna-1,4-diene-11β,17α,21-triol-3,20-dione (6α-Methyl-prednisolone)  6α-Methyl-pregna-1,4-diene-11β,17α,20β,21-tetraol-3-one (20β-Reduced 6α-Methyl-prednisolone) | - | [38] |
| 6α-Methyl-pregna-4,6-diene-11β,17α,21-triol-3,20-dione (6(7)-Dehydro-6α-methyl-hydrocortisone) | 6α-Methyl-pregna-1,4,6-triene-11β,17α,21-triol-3,20-dione (6(7)-Dehydro-6α-methyl-prednisolone,  6α-Methyl-pregna-1,4,6-triene-11β,17α,20β,21-tetraol-3-one (20β-Reduced 6(7)-Dehydro-6α-methyl-prednisolone) | - | [98]* |
| 16α-Methyl-pregn-4-ene-11β,17α,21-triol-3,20-dione (16α-Methyl-hydrocortisone) | 16α-Methyl-pregna-1,4-diene-11β,17α,21-triol-3,20-dione  (16α-Methyl-prednisolone) | - | [33] |
| Androst-4-ene-3,17-dione (AD) | Androsta-1,4-diene-3,17-dione (ADD)  Androst-4-en-17β-ol-3-one (Testosterone, T)  Androsta-1,4-dien-17β-ol-3-one (1(2)-Dehydro-testosterone, Boldenone, DT) | + | [34] |
| Androst-4-en-17β-ol-3-one  (Testosterone, T) | Androsta-1,4-dien-17β-ol-3-one~~,~~ DT)  AD  ADD | + | [34] |
| 6(α/β)-(*N*-methyl-*N*-phenyl)aminomethylandrost-4-ene-3,17-dione  (6(α/β)-(*N*-methyl-*N*-phenyl)aminomethyl-AD | 6β-(*N*-methyl-*N*-phenyl)aminomethylandrost-4-en-17β-ol-3-one  (6β-(*N*-methyl-*N*-phenyl)aminomethyl-T)  6β-(*N*-methyl-*N*-phenyl)aminomethylandrosta-1,4-dien-17β-ol-3-one (6β-(*N*-methyl-*N*-phenyl)aminomethyl-DT)  6β-(*N*-methyl-*N*-phenyl)aminomethylandrosta-1,4-diene-3,17-dione (6β-(*N*-methyl-*N*-phenyl)aminomethyl-ADD) | - | [34] |
| 6(α/β)-(*N*-methyl-*N*-phenyl)aminomethylandrost-4-en-17β-ol-3-one (6β-(*N*-methyl-*N*-phenyl)aminomethyl-T) | 6(α/β)-(*N*-methyl-*N*-phenyl)aminomethyl-androst-4-ene-3,17-dione (6(α/β)-(*N*-methyl-*N*-phenyl)aminomethyl-AD)  6β-(*N*-methyl-*N*-phenyl)aminomethyl-androsta-1,4-dien-17β-ol-3-one (6β-(*N*-methyl-*N*-phenyl)aminomethyl-DT)  6β-(*N*-methyl-*N*-phenyl)aminomethyl-androsta-1,4-diene-3,17-dione (6β-(*N*-methyl-*N*-phenyl)aminomethyl-ADD) | - | [34] |
| 6-Methylene-androst-4-ene-3,17-dione  (6-Methylene-AD) | 6-Methylene-androsta-1,4-diene-3,17-dione (6-Methylene-ADD) | - | [39] |
| 17α-Methyl-androst-4-en-17β-ol-3-one (17α-Methyl-T) | 17α-Methyl-Androsta-1,4-dien-17β-ol-3-one (17α-Methyl-DT) | + | [39] |
| Androsta-4,9-diene-3,17-dione  (Δ^9^-AD) | Androsta-1,4,9-triene-3,17-dione  (Δ^9^-ADD) | - | [39] |
| 21-Acetoxy-pregna-4(5),9(11),16(17)-triene-21-ol-3,20-dione | 21-Acetoxy-pregna-1(2),4(5),9(11),16(17)-tetraene-21-ol-3,20-dione  Pregna-1(2),4(5),9(11),16(17)-tetraene-21-ol-3,20-dione | - | [32] |
| Pregna-4,9(11)-diene-17α,21-diol-3,20-dione 21-acetate | Pregna-1,4,9(11)-triene-17α,21-diol-3,20-dione 21-acetate  Pregna-1,4,9(11)-triene-17α,21-diol-3,20-dione  Pregna-4,9(11)-diene-17α,21-diol-3,20-dione  Pregna-1,4,9(11)-triene-17α,20β,21-triol-3-one | - | [31] |
| Pregna-4,9(11)-diene-17α,21-diol-3,20-dione 17,21-diacetate | Pregna-1,4,9(11)-triene-17α,21-diol-3,20-dione 17,21-diacetate  Pregna-1,4,9(11)-triene-17α,21-diol-3,20-dione 21-acetate  Pregna-1,4,9(11)-triene-17α,21-diol-3,20-dione  Pregna-1,4,9(11)-triene-17α,21-diol-3,20-dione 17-acetate  Pregna-1,4,9(11)-triene-17α,20β,21-triol-3-one | - | [31] |
| Pregn-4-ene-3,20-dione (Progesterone) | Pregna-1,4-diene-3,20-dione (1(2)-Dehydro-progesterone) | + | This work |
| Pregn-4-en-17α-ol-3,20-dione  (17α-Hydroxy-progesterone) | Pregna-1,4-dien-17α-ol-3,20-dione (1(2)-Dehydro-17α-hydroxy-progesterone) | + | This work |
| Pregn-4-ene-11α,17α-diol-3,20-dione  (11α,17α-Dihydroxy-progesterone) | (Pregna-1,4-diene-11α,17α-diol-3,20-dione (1(2)-Dehydro-11α,17α-dihydroxy-progesterone) | + | This work |

* Citations 97 and 98 are not included in the body of the article.

[97] Lobastova TG, Fokina VV, Sukhodolskaya GV, Shutov AA, Donova MV. Transformation of C-19, C-21 steroids and cholic acids by Nocardioids simplex VKM Ac-2033D. In: Vth Pushchino school-conference “Biochemistry, physiology and the biosphere role of microorganisms.” Moscow; Publisher: "Water: chemistry and ecology", 2018. 176 p. ISBN 978-5-9909335-6-9. p. 114–116.

[98] Savinova TS, Fokina VV, Gainova KM, Kazantsev AV, Donova MV, Lukashev NV. New synthesis of 6-dehydro-analogue of 6-methylprednisolone from 21-acetate hydrocortisone by chemical-biotechnological method. In: V-th All-Russian Conference on organic chemistry with International participation; Vladikavkaz, Republic of North Ossetia – Alania. Publisher: North Ossetian State University named after K.L. Khetagurov, 2018. 554 p. p. 385

Supplementary table S2 MS-characteristics of the steroid substrates and *N. simplex* bioconversion products

| *Name of substances* | *Short name* | *M.w.* | *General formula* | *MS/MS (intensity, %) [M+H]^+^* |
| --- | --- | --- | --- | --- |
| Substrates | | | | |
| Progesterone | Pr | 314.5 | C_21_H_30_O_2_ | 23/315 (100), 297 (95), 279 (23), 253 (5), 215 (8), 123 (5); 97 (15) |
| 17α-Hydroxyprogesterone | 17α-OH-Pr | 330.5 | C_21_H_30_O_3_ | 23/331 (100), 313 (70), 295 (35), 285 (14), 277 (15), 271 (14), 253 (10), 97 (10) |
| 11α,17α-Dihydroxyprogesterone | 11α,17α-di-OH-Pr | 346.5 | C_21_H_30_O_4_ | 22/347 (62), 329 (48), 311 (100), 293 (20), 283 (18), 269 (8) |
| 21-Acetate of cortisone | AcC | 402.5 | C_23_H_30_O_6_ | 17/403 (50), 385 (100), 361 (10), 343 (20); 325(17) |
| β-Sitosterol |  | 414.7 | C_29_H_50_O | 26/415 (66), 414 (56), 397 (29), 396 (100) |
| Stigmasterol |  | 412.7 | C_29_H_48_O | 26/413 (45), 395 (100), 297 (11), 283 (10), 255 (12), 241 (10) |
| Campesterol |  | 400.7 | C_28_H_48_O | 27/401 (52), 400 (85), 383 (57), 382 (100) |
| Brassicasterol |  | 398.7 | C_28_H_46_O | 399 |
| Products | | | | |
| 1(2)-Dehydro-progesterone | DPr | 312.5 | C_21_H_28_O_2_ | 22/313 (65), 295 (100), 277 (5), 253 (5), 217 (10), 179 (14), 121 (5) |
| 1(2)-Dehydro-17α-hydroxyprogesterone | 17α-OH-DPr | 328.5 | C_21_H_28_O_3_ | 21/329 (80), 311 (100), 293 (20), 283 (21), 273 (8), 121 (5) |
| 1(2)-Dehydro-11α,17α-dihydroxyprogesterone | 11α,17α-di-OH-DPr | 344.5 | C_21_H_28_O_4_ | 19/345 (84), 327 (100), 309 (22), 291 (5), 281 (5) |
| Cortisone | C | 360.5 | C_21_H_28_O_5_ | 24/361 (50), 343 (100), 325 (45), 313 (15), 307 (20), 301 (23), 295 (8), 283 (28), 265 (25), 163 (45), 121 (5) |
| 21-Acetate of prednisone | AcP, or 1(2)-dehydro-AcC | 400.5 | C_23_H_28_O_6_ | 18/401 (65), 383 (100), 359 (5), 341 (25), 323 (8), 305 (5), 295 (10) |
| Prednisone | P, or 1(2)-dehydro-cortisone | 358.5 | C_21_H_26_O_5_ | 19/359 (56), 341 (100), 323 (25), 313 (25), 305 (11), 295 (10), 281 (5) |
| 20β-reduced cortisone | 20β-ОН-C | 362.5 | C_21_H_30_O_5_ | 17/361 (95), 343 (100), 325 (18), 313 (15), 307 (10), 297 (3), 295 (2), 283 (8), 265 (5), 161 (5) |
| 20β-reduced prednisone | 20β-ОН-P | 360.5 | C_21_H_28_O_5_ | 22/359 (45), 329 (100), 311 (5) |
| Stigmast-4-en-3-one  (β-sitostenone) |  | 412.7 | C_29_H_48_O | 26/413 (80), 412 (75), 395 (100), 394 (15); 123 (2) |
| Stigmasta-4,22-dien-3-one |  | 410.7 | C_29_H_46_O | 27/411 (100), 393 (73), 327 (87), 309 (30), 295 (13), 271 (60), 123 (1) |
| Campest-4-en-3-one |  | 398.7 | C_28_H_46_O | 30/399 (18), 398 (100), 381 (75), 316 (58); 258 (75), 244 (82), 161 (51), 135 (13) |
| Brassicast-4-en-3-one |  | 396.7 | C_28_H_44_O | 28/397 (100), 379 (32), 315 (56), 257 (75), 243 (93), 161 (75), 135 (43) |

***Supplementary table S3 ^1^H-NMR spectra***

| *Spectrum* | *Substance and its characteristics* | |
| --- | --- | --- |
| 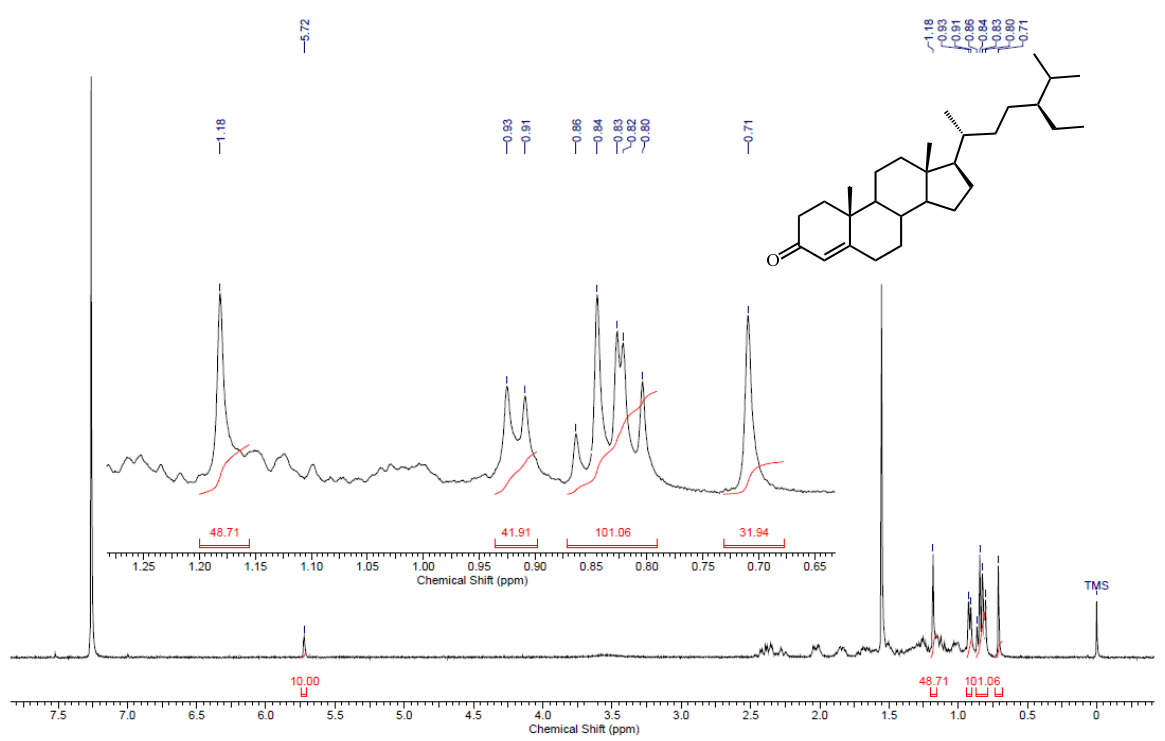 | Stigmast-4-en-3-one  (β-sitostenone)^1^H-NMR (CDCl_3_) δ: 5.72 (s, 1H, 4-H), 1.18 (s, 3H, 19-CH_3_), 0.92 (d, J = 6.5 Hz, 3H, 21-CH_3_), 0.84 (t, J = 7.2 Hz, 3H, 29-CH_3_), 0.83 (d, J = 6.9 Hz, 3H, 26(27)-CH_3_), 0.81 (d, J = 6.9 Hz, 3H, 26(27)-CH_3_), 0.71 (s, 3H, 18-CH_3_). | |
| 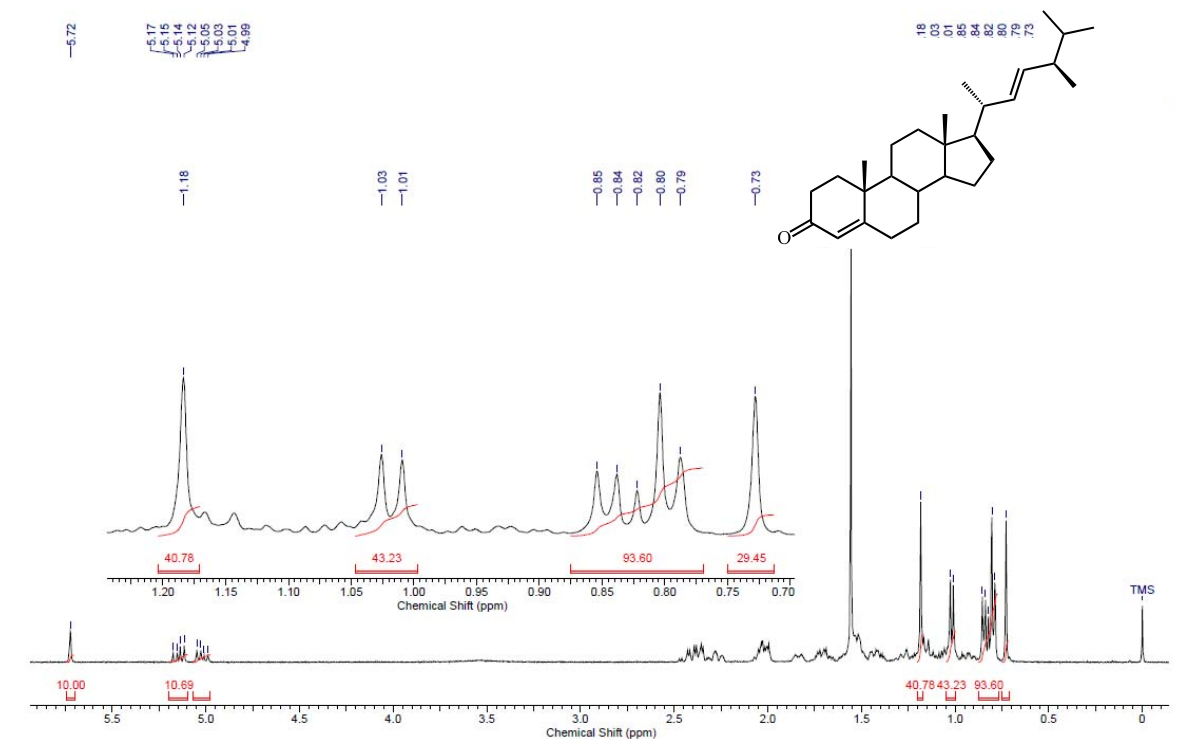 | | Brassicast-4-en-3-one ^1^H-NMR (CDCl_3_) δ: 5.72 (s, 1H, 4-H), 5.11-5.18 (m, 1H, 22(23)-H), 4.98-5.06 (m, 1H, 22(23)-H), 1.18 (s, 3H, 19-CH_3_), 1.02 (d, J = 6.6 Hz, 3H, 21-CH_3_), 0.84 (d, J = 6.3 Hz, 3H, 28-CH_3_), 0.81 (d, J = 6.9 Hz, 3H, 26(27)-CH_3_), 0.79 (d, J = 6.9 Hz, 3H, 26(27)-CH_3_), 0.73 (s, 3H, 18-CH_3_). |

**Supplementary Table S5** New candidate motifs for binding of transcription factors for steroid catabolism regulation in *N. simplex* VKM Ac-2033D

| Motif | *E*-value | LOGO | Genes with this motif (MEME) | Genes with this motif (FIMO) |
| --- | --- | --- | --- | --- |
| Acin1 | 8.6*10^-6^ | 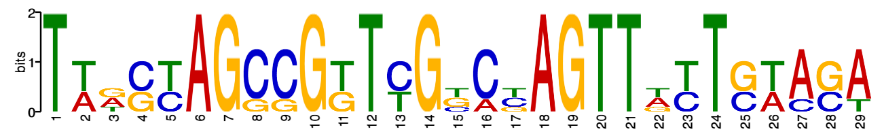 | *KR76_27130* and  *KR76_27125*  *KR76_24100*  *KR76_24405* and  *KR76_24400* | *KR76_24105* and *KR76_24100*  *KR76_24405* and  *KR76_24400*  *KR76_26730*  *KR76_27060*  *KR76_27130* and  *KR76_27125* |
| Acin2 | 0.0083 | 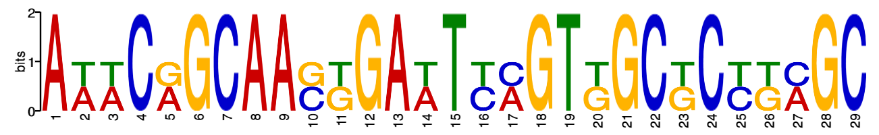 | *KR76_16075* and *KR76_16070* | *KR76_16075* and *KR76_16070*  *KR76_1391* |
| Acin3 | 0.095 | 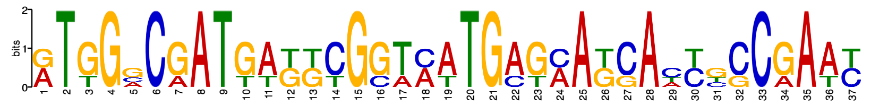 | *KR76_26475*  *KR76_27130* and *KR76_27125*  *KR76_27375* | *KR76_25695*  *KR76_26470* and *KR76_26475*  *KR76_27130* and *KR76_27125*  *KR76_27380* and *KR76_27375* |
| Sitdec1 | 0.0093 | 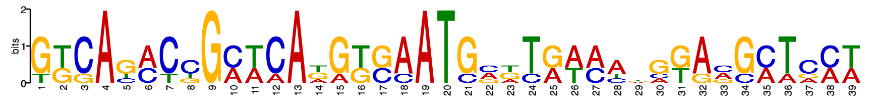 | *KR76_00525* and *KR76_00520*  *KR76_03130*  *KR76_12475*  *KR76_22685* | *KR76_00225* and *KR76_00220*  *KR76_08640*  *KR76_09040*  *KR76_12470* and *KR76_12475*  *KR76_19130*  *KR76_22680* and *KR76_22685*  *KR76_26570* and *KR76_26565* |

**Supplementary table S6** Real-time qPCR

Primers for real-time Q-PCR

| Gene | F | R | Tm | Length |
| --- | --- | --- | --- | --- |
| *IF 1 KR76_21135* | ATGTGAGCCAGGACCTTGTG | ATGGCGAAGAAAGAAGGCGT | 60 | 110 |
| *gyrA KR76_00045* | ACTGAAACCCAGAGCAACCT | TGGCGTAGTCGATGTAGGAG | 60 | 100 |
| *petA KR76_10970* | GCTCATAGTAGTCACGCTCCC | ACTCGAAGATCTGCACCCAC | 60 | 246 |
| *cyp125 KR76_14335* | TGGACCACTCGAACAGCTTC | TCCCGACATCAACGAAGTGG | 60 | 503 |
| *fadD3 KR76_25120* | GACATCCTCTTCACCTCGGG | CTTGTAGCCGAAGCTGTGGA | 60 | 162 |

Expression changes (2^-ΔΔCt^) for target genes

| Gene | Phytosterol induction | AcC induction |
| --- | --- | --- |
| *cyp125 KR76_14335* | 5.7 | 0.9 |
| *fadD3 KR76_25120* | 2.4 | 0.2 |
