# Supplementary material for: Different genome-wide transcriptome responses of Nocardioides simplex VKM Ac-2033D to phytosterol and cortisone 21-acetate
Source: BMC Biotechnol. 2021 Jan 13;21:7. doi: 10.1186/s12896-021-00668-9 (PMC7807495; doi:10.1186/s12896-021-00668-9)
Supplement: Supplementary file 3 — Additional file 3: Supplementary Figure S1-S3. Alignments of KshA, KshB, KstD [pdf]. Supplementary Figure S1. KshA alignment. Supplementary Figure S2. KshB alignment. Supplementary Figure S3. KstD alignment. [file 12896_2021_668_MOESM3_ESM.pdf]

Different genome-wide transcriptome responses of *Nocardioides simplex* VKM Ac-2033D to phytosterol and cortisone-21-acetate

Victoria Yu. Shtratnikova[1], Mikhail I. Schelkunov[2,3], Victoria V. Fokina[4,5], Eugeny Y. Bragin[4], Andrei A. Shutov[4,5], Marina V. Donova[4,5].

[1]Belozersky Institute of Physico-Chemical Biology, Lomonosov Moscow State University, Leninskie gory, h. 1, b. 40, Moscow, Russian Federation, 119991

[2]Skolkovo Institute of Science and Technology, Nobelya str., 3, Moscow, Russian Federation, 121205

[3]Institute for Information Transmission Problems, Russian Academy of Sciences, Bolshoy Karetny per., h. 19, b. 1, Moscow, Russian Federation, 127994

[4]G.K. Skryabin Institute of Biochemistry and Physiology of Microorganisms, Federal Research Center “Pushchino Center for Biological Research of the Russian Academy of Sciences”, pr. Nauki, 5, Pushchino, Moscow Region, Russian Federation, 142290

[5]Pharmins, Ltd., R&D, Institutskaya str., 4, Pushchino, Moscow Region, Russian Federation, 142290

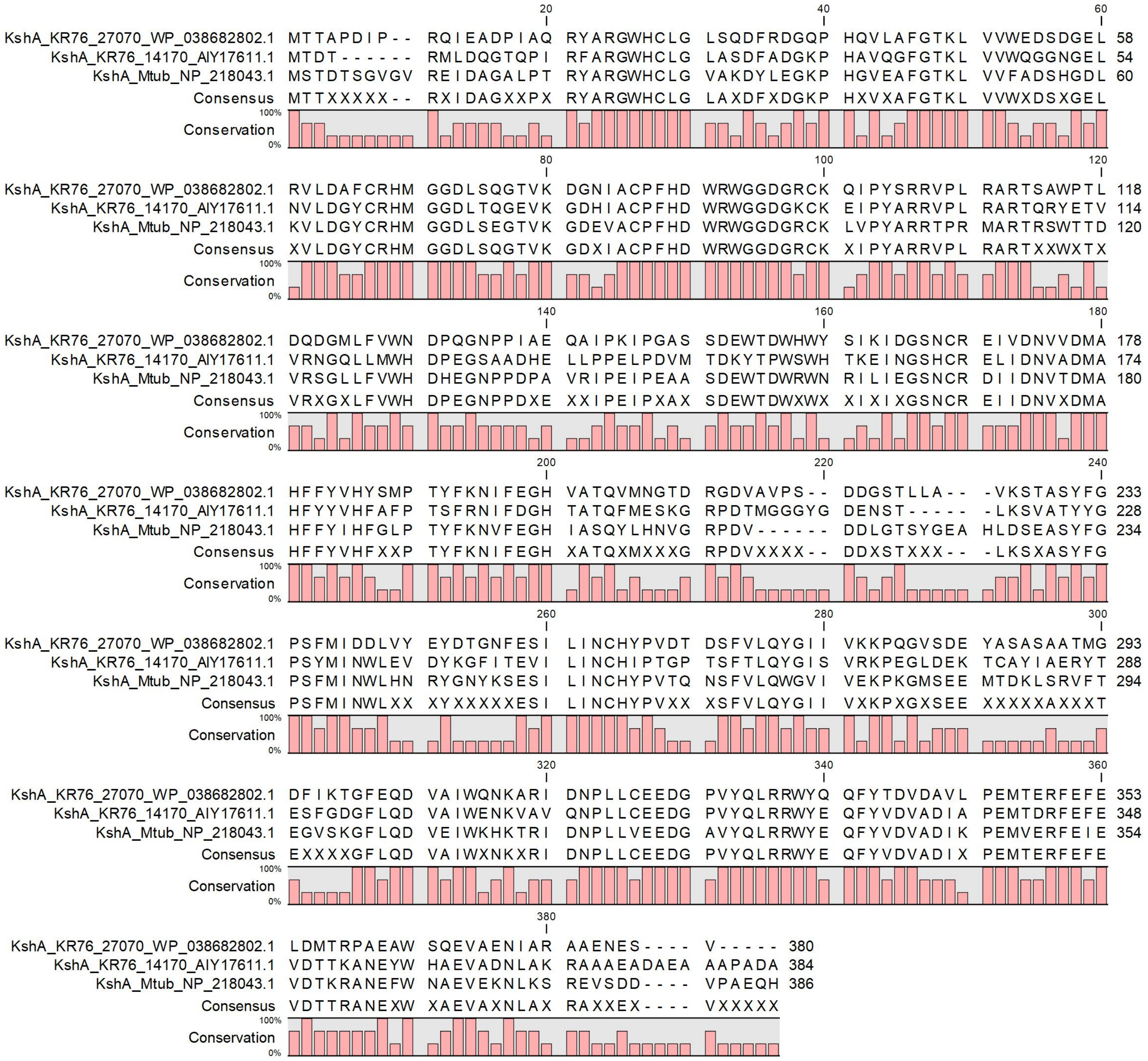
KshA_KR76_27070_WP_038682802.1 MTTAPDIP-- RQIEADPIAQ RYARGWHCLG LSQDFRDGQP HQVLAFGTKL VVWEDSDGEL 58
KshA_KR76_14170_AIY17611.1 MTDT------ RMLDQGTQPI RFARGWHCLG LASDFADGKP HAVQGFGTKL VVWQGGNGEL 54
KshA_Mtub_NP_218043.1 MSTDTSGVGV REIDAGALPT RYARGWHCLG VAKDYLEGKP HGVEAFGTKL VVFADSHGDL 60
Consensus MTTXXXXX-- RXIDAGXXPX RYARGWHCLG LAXDFXDGKP HXVXAFGTKL VVWXDSXGEL
Conservation
KshA_KR76_27070_WP_038682802.1 RVLDAFCRHM GGDLSQGTVK DGNIACPFHD WRWGGDGRCK QIPYSRRVPL RARTSAWPTL 118
KshA_KR76_14170_AIY17611.1 NVLDGYCRHM GGDLTQGEVK GDHIACPFHD WRWGGDGKCK EIPYARRVPL RARTQRYETV 114
KshA_Mtub_NP_218043.1 KVLDGYCRHM GGDLSEGTVK GDEVACPFHD WRWGGDGRCK LVPYARRTPR MARTRSWTTD 120
Consensus XVLDGYCRHM GGDLSQGTVK GDXIACPFHD WRWGGDGRCK XIPYARRVPL RARTXXWXTX
Conservation
KshA_KR76_27070_WP_038682802.1 DQDGMLFVWN DPQGNPPIAE QAIPKIPGAS SDEWTDWHWY SIKIDGSNCR EIVDNVVDMA 178
KshA_KR76_14170_AIY17611.1 VRNGQLLMWH DPEGSAADHE LLPPELPDVM TDKYTPWSWH TKEINGSHCR ELIDNVADMA 174
KshA_Mtub_NP_218043.1 VRSGLLFVWH DHEGNPPDPA VRIPEIPEAA SDEWTDWRWN RILIEGSNCR DIIDNVTDMA 180
Consensus VRXGXLFVWH DPEGNPPDXE XXIPEIPXAX SDEWTDWXWX XIXIXGSNCR EIIDNVXDMA
Conservation
KshA_KR76_27070_WP_038682802.1 HFFYVHYSMP TYFKNIFEGH VATQVMNGTD RGDVAVPS-- DDGSTLLA-- -VKSTASYFG 233
KshA_KR76_14170_AIY17611.1 HFYYVHFAFP TSFRNIFDGH TATQFMESKG RPDTMGGGYG DENST----- -LKSVATYYG 228
KshA_Mtub_NP_218043.1 HFFYIHFGLP TYFKNVFEGH IASQYLHNVG RPDV------ DDLGTSYGEA HLDSEASYFG 234
Consensus HFFYVHFXXP TYFKNIFEGH XATQXMXXXG RPDVXXXX-- DDXSTXXX-- -LKSXASYFG
Conservation
KshA_KR76_27070_WP_038682802.1 PSFMIDDLVY EYDTGNFESI LINCHYPVDT DSFVLQYGII VKKPQGVSDE YASASAATMG 293
KshA_KR76_14170_AIY17611.1 PSYMINWLEV DYKGFITEVI LINCHIPTGP TSFTLQYGIS VRKPEGLDEK TCAYIAERYT 288
KshA_Mtub_NP_218043.1 PSFMINWLHN RYGNYKSESI LINCHYPVTQ NSFVLQWGVI VEKPKGMSEE MTDKLSRVFT 294
Consensus PSFMINWLXX XYXXXXXESI LINCHYPVXX XSFVLQYGII VXKPXGXSEE XXXXXAXXXT
Conservation
KshA_KR76_27070_WP_038682802.1 DFIKTGFEQD VAIWQNKARI DNPLLCEEDG PVYQLRRWYQ QFYTDVDAVL PEMTERFEFE 353
KshA_KR76_14170_AIY17611.1 ESFGDGFLQD VAIWENKVAV QNPLLCEEDG PVYQLRRWYE QFYVDVADIA PEMTDRFEFE 348
KshA_Mtub_NP_218043.1 EGVSKGFLQD VEIWKHKTRI DNPLLVEEDG AVYQLRRWYE QFYVDVADIK PEMVERFEIE 354
Consensus EXXXXGFLQD VAIWXNKXRI DNPLLCEEDG PVYQLRRWYE QFYVDVADIX PEMTERFEFE
Conservation
KshA_KR76_27070_WP_038682802.1 LDMTRPAEAW SQEVAENIAR AAENES---- V----- 380
KshA_KR76_14170_AIY17611.1 VDTTKANEYW HAEVADNLAK RAAAEADAEA AAPADA 384
KshA_Mtub_NP_218043.1 VDTKRANEFW NAEVEKNLKS REVSDD---- VPAEQH 386
Consensus VDTTRANEXW XAEVAXNLAX RAXXEX---- VXXXXX
Conservation

Supplementary Figure S1. KshA alignment

KshA from *M. tuberculosis* consists of conservative Rieske domain (amino acid residues 24-153), where C67 and C86 are responsible for one Fe^{2+} ion binding, and H69 and H89 — for another [71]. The catalytic domain (amino acid residues 154-374) contains conserved amino acid residues V176, D178, H181, H186, Q204, Y232, M238, N240, N257, F301, D304 and W308. In *KR76_27070* of *N. simplex*, N240 is replaced with D240 in this protein.

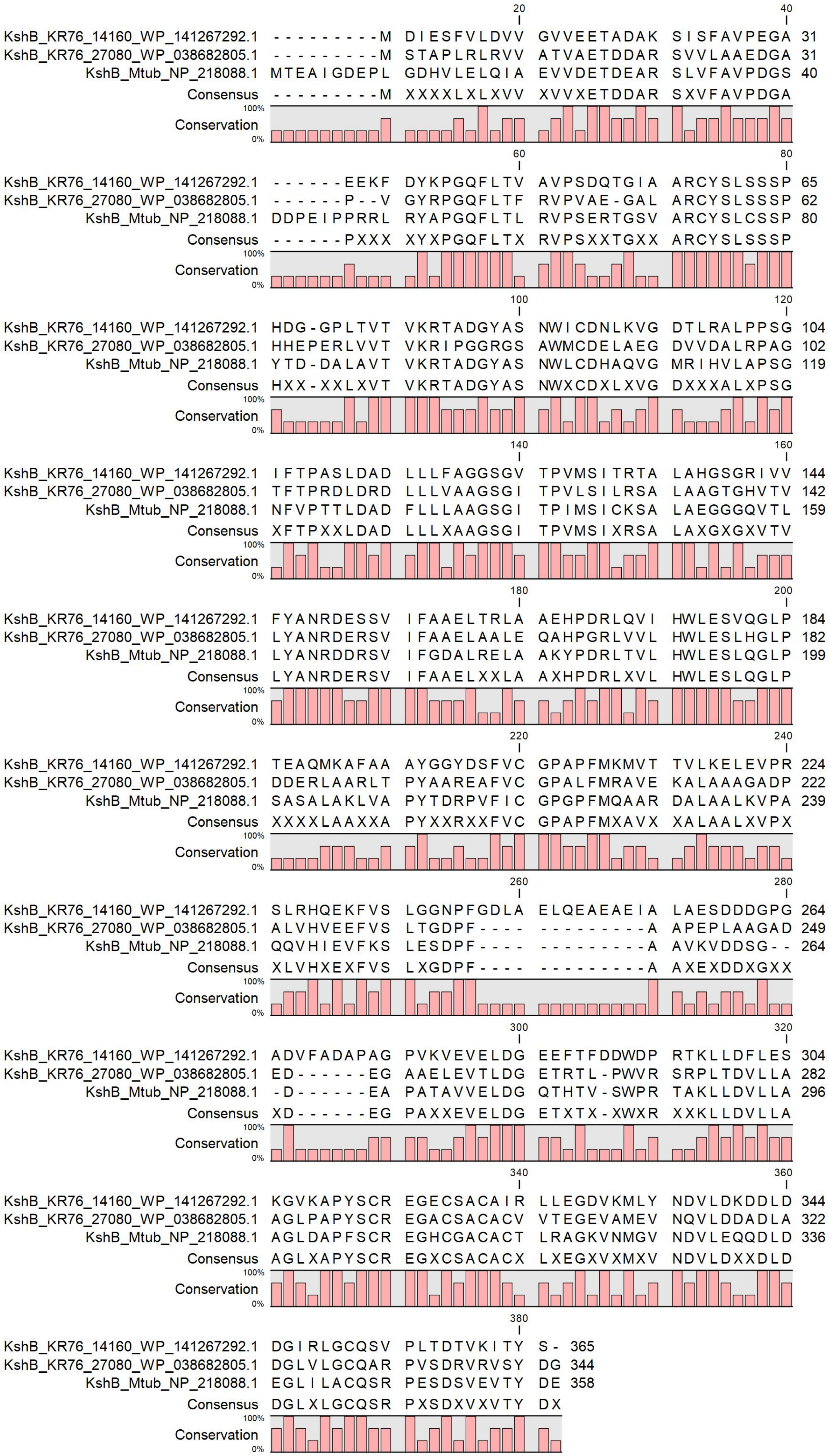
20
40
KshB_KR76_14160_WP_141267292.1 ---------M DIESFVLDVV GVVEETADAK SISFAVPEGA 31
KshB_KR76_27080_WP_038682805.1 ---------M STAPLRLRVV ATVAETDDAR SVVLAAEDGA 31
KshB_Mtub_NP_218088.1 MTEAIGDEPL GDHVLELQIA EVVDETDEAR SLVFAVPDGS 40
Consensus ---------M XXXXLXLXVV XVVXETDDAR SXVFAVPDGA
100%
Conservation
0%
60
80
KshB_KR76_14160_WP_141267292.1 ------EEKF DYKPGQFLTV AVPSDQTGIA ARCYSLSSSP 65
KshB_KR76_27080_WP_038682805.1 ------P--V GYRPGQFLTF RVPVAE-GAL ARCYSLSSSP 62
KshB_Mtub_NP_218088.1 DDPEIPPRRL RYAPGQFLTL RVPSERTGSV ARCYSLCSSP 80
Consensus ------PXXX XYXPGQFLTX RVPSXXTGXX ARCYSLSSSP
100%
Conservation
0%
100
120
KshB_KR76_14160_WP_141267292.1 HDG-GPLTVT VKRTADGYAS NWICDNLKVG DTLRALPPSG 104
KshB_KR76_27080_WP_038682805.1 HHEPERLVVT VKRIPGGRGS AWMCDELAEG DVVDALRPAG 102
KshB_Mtub_NP_218088.1 YTD-DALAVT VKRTADGYAS NWLCDHAQVG MRIHVLAPSG 119
Consensus HXX-XXLXVT VKRTADGYAS NWXCDXLXVG DXXXALXPSG
100%
Conservation
0%
140
160
KshB_KR76_14160_WP_141267292.1 IFTPASLDAD LLLFAGGSGV TPVMSITRTA LAHGSGRIVV 144
KshB_KR76_27080_WP_038682805.1 TFTPRDLDRD LLLVAAGSGI TPVLSILRSA LAAGTGHVTV 142
KshB_Mtub_NP_218088.1 NFVPTTLDAD FLLLAAGSGI TPIMSICKSA LAEGGGQVTL 159
Consensus XFTPXXLDAD LLLXAAGSGI TPVMSIXRSA LAXGXGXVTV
100%
Conservation
0%
180
200
KshB_KR76_14160_WP_141267292.1 FYANRDESSV IFAAELTRLA AEHPDRLQVI HWLESVQGLP 184
KshB_KR76_27080_WP_038682805.1 LYANRDERSV IFAAELAALE QAHPGRLVVL HWLESLHGLP 182
KshB_Mtub_NP_218088.1 LYANRDDRSV IFGDALRELA AKYPDRLTVL HWLESLQGLP 199
Consensus LYANRDERSV IFAAELXXLA AXHPDRLXVL HWLESLQGLP
100%
Conservation
0%
220
240
KshB_KR76_14160_WP_141267292.1 TEAQMKAFAA AYGGYDSFVC GPAPFMKMVT TVLKELEVPR 224
KshB_KR76_27080_WP_038682805.1 DDERLAARLT PYAAREAFVC GPALFMRAVE KALAAAGADP 222
KshB_Mtub_NP_218088.1 SASALAKLVA PYTDRPVFIC GPGPFMQAAR DALAALKVPA 239
Consensus XXXXLAAXXA PYXXRXXFVC GPAPFMXAVX XALAALXVPX
100%
Conservation
0%
260
280
KshB_KR76_14160_WP_141267292.1 SLRHQEKFVS LGGNPFGDLA ELQEAEAEIA LAESDDDGPG 264
KshB_KR76_27080_WP_038682805.1 ALVHVEEFVS LTGDPF---- ---------A APEPLAAGAD 249
KshB_Mtub_NP_218088.1 QQVHIEVFKS LESDPF---- ---------A AVKVDDSG-- 264
Consensus XLVHXEXFVS LXGDPF---- ---------A AXEXDDXGXX
100%
Conservation
0%
300
320
KshB_KR76_14160_WP_141267292.1 ADVFADAPAG PVKVEVELDG EEFTFDDWDP RTKLLDFLES 304
KshB_KR76_27080_WP_038682805.1 ED------EG AAELEVTLDG ETRTL-PWVR SRPLTDVLLA 282
KshB_Mtub_NP_218088.1 -D------EA PATAVVELDG QTHTV-SWPR TAKLLDVLLA 296
Consensus XD------EG PAXXEVELDG ETXTX-XWXR XXKLLDVLLA
100%
Conservation
0%
340
360
KshB_KR76_14160_WP_141267292.1 KGVKAPYSCR EGECSACAIR LLEGDVKMLY NDVLDKDDLD 344
KshB_KR76_27080_WP_038682805.1 AGLPAPYSCR EGACSACACV VTEGEVAMEV NQVLDDADLA 322
KshB_Mtub_NP_218088.1 AGLDAPFSCR EGHCGACACT LRAGKVNMGV NDVLEQQDLD 336
Consensus AGLXAPYSCR EGXCSACACX LXEGXVXMXV NDVLDXXDLD
100%
Conservation
0%
380
KshB_KR76_14160_WP_141267292.1 DGIRLGCQSV PLTDTVKITY S- 365
KshB_KR76_27080_WP_038682805.1 DGLVLGCQAR PVSDRVRVSY DG 344
KshB_Mtub_NP_218088.1 EGLILACQSR PESDSVEVTY DE 358
Consensus DGLXLGCQSR PXSDXVXVTY DX
100%
Conservation
0%

Supplementary Figure S2. KshB alignment

There are three domains in KshB containing a number of conservative amino acid residues, namely, a flavin binding domain RxYSL (72-76 in product of Rv3571 *M. tuberculosis*), a NAD binding domain GGIGITP or AGSGITP (135-141 in product of Rv3571 *M. tuberculosis*), and a 2Fe2S-cluster binding domain $Cx_4Cx_2Cx_{29}C$ (305-344 in product of Rv3571 *M. tuberculosis*) [85]. All three domains were also found in ortholog of KshB in *N. simplex* with two

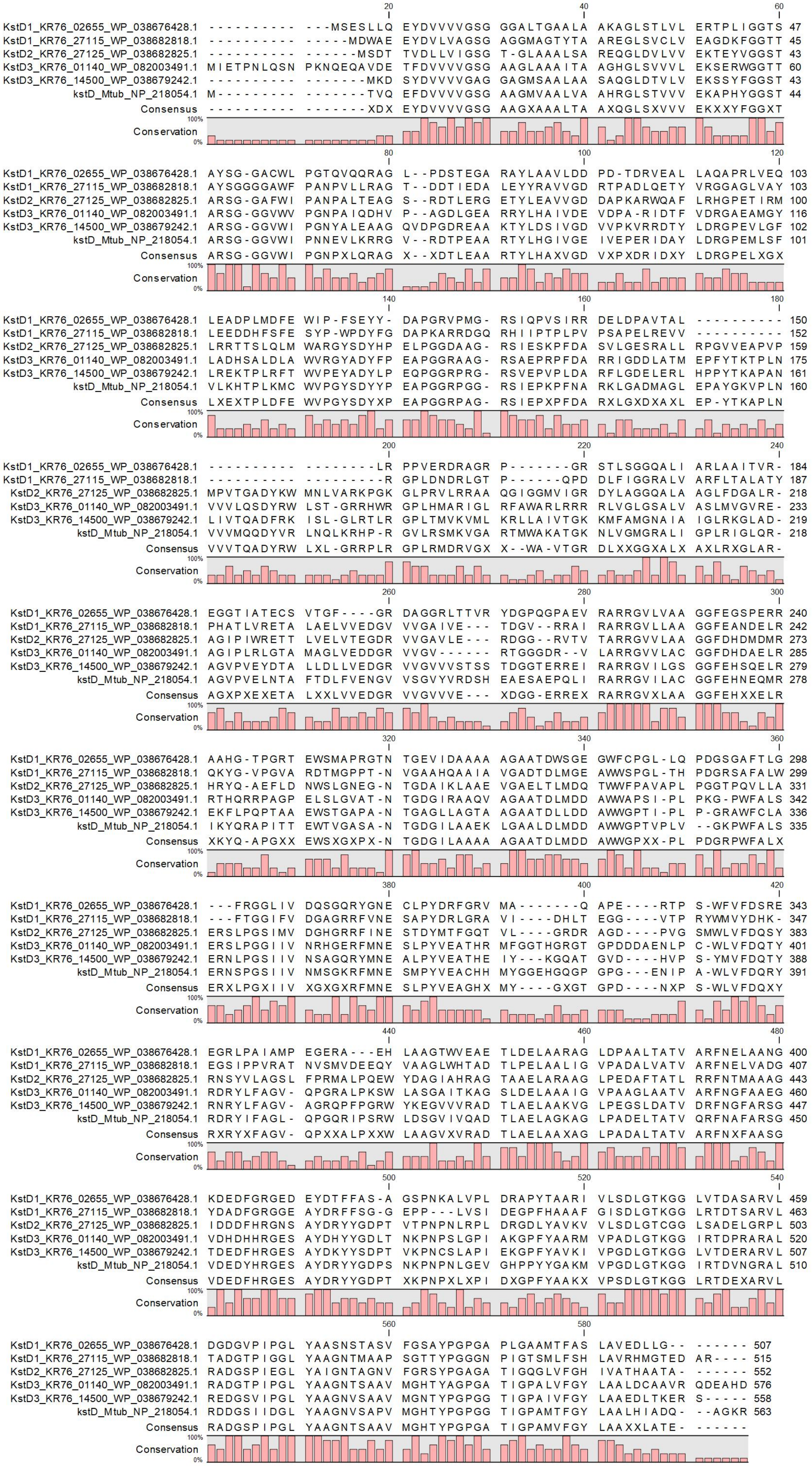
20 40 60
KstD1_KR76_02655_WP_038676428.1 ---------- ---MSESLLQ EYDVVVVGSG GGALTGAALA AKAGLSTLVL ERTPLIGGTS 47
KstD1_KR76_27115_WP_038682818.1 ---------- -----MDWAE EYDVLVAGSG AGGMAGTYTA AREGLSVCLV EAGDKFGGTT 45
KstD2_KR76_27125_WP_038682825.1 ---------- ------MSDT TVDLLVIGSG T-GLAAALSA REQGLDVLVV EKTEYVGGST 43
KstD3_KR76_01140_WP_082003491.1 MIETPNLQSN PKNQEQAVDE TFDVVVVGSG AAGLAAAITA AGHGLSVVVL EKSERWGGTT 60
KstD3_KR76_14500_WP_038679242.1 ---------- -------MKD SYDVVVVGAG GAGMSAALAA SAQGLDTVLV EKSSYFGGST 43
kstD_Mtub_NP_218054.1 M--------- -------TVQ EFDVVVVGSG AAGMVAALVA AHRGLSTVVV EKAPHYGGST 44
Consensus ---------- -------XDX EYDVVVVGSG AAGXAAALTA AXQGLSXVVV EKXXYFGGXT
Conservation 100% 0%
80 100 120
KstD1_KR76_02655_WP_038676428.1 AYSG-GACWL PGTQVQQRAG L--PDSTEGA RAYLAAVLDD PD-TDRVEAL LAQAPRLVEQ 103
KstD1_KR76_27115_WP_038682818.1 AYSGGGGAWF PANPVLLRAG T--DDTIEDA LEYYRAVVGD RTPADLQETY VRGGAGLVAY 103
KstD2_KR76_27125_WP_038682825.1 ARSG-GAFWI PANPALTEAG S--RDTLERG ETYLEAVVGD DAPKARWQAF LRHGPETIRM 100
KstD3_KR76_01140_WP_082003491.1 ARSG-GGVWV PGNPAIQDHV P--AGDLGEA RRYLHAIVDE VDPA-RIDTF VDRGAEAMGY 116
KstD3_KR76_14500_WP_038679242.1 ARSG-GGVWI PGNYALEAAG QVDPGDREAA KTYLDSIVGD VVPKVRRDTY LDRGPEVLGF 102
kstD_Mtub_NP_218054.1 ARSG-GGVWI PNNEVLKRRG V--RDTPEAA RTYLHGIVGE IVEPERIDAY LDRGPEMLSF 101
Consensus ARSG-GGVWI PGNPXLQRAG X--XDTLEAA RTYLHAXVGD VXPXDRIDXY LDRGPELXGX
Conservation 100% 0%
140 160 180
KstD1_KR76_02655_WP_038676428.1 LEADPLMDFE WIP-FSEYY- DAPGRVPMG- RSIQPVSIRR DELDPAVTAL ---------- 150
KstD1_KR76_27115_WP_038682818.1 LEEDDHFSFE SYP-WPDYFG DAPKARRDGQ RHIIPTPLPV PSAPELREVV ---------- 152
KstD2_KR76_27125_WP_038682825.1 LRRTTSLQLM WARGYSDYHP ELPGGDAAG- RSIESKPFDA SVLGESRALL RPGVVEAPVP 159
KstD3_KR76_01140_WP_082003491.1 LADHSALDLA WVRGYADYFP EAPGGRAAG- RSAEPRPFDA RRIGDDLATM EPFYTKTPLN 175
KstD3_KR76_14500_WP_038679242.1 LREKTPLRFT WVPEYADYLP EQPGGRPRG- RSVEPVPLDA RFLGDELERL HPPYTKAPAN 161
kstD_Mtub_NP_218054.1 VLKHTPLKMC WVPGYSDYYP EAPGGRPGG- RSIEPKPFNA RKLGADMAGL EPAYGKVPLN 160
Consensus LXEXTPLDFE WVPGYSDYXP EAPGGRPAG- RSIEPXPFDA RXLGXDXAXL EP-YTKAPLN
Conservation 100% 0%
200 220 240
KstD1_KR76_02655_WP_038676428.1 ---------- --------LR PPVERDRAGR P-------GR STLSGGQALI ARLAAITVR- 184
KstD1_KR76_27115_WP_038682818.1 ---------- ---------- GPLDNDRLGT P-------QPD DLFIGGRALV ARFLTALATY 187
KstD2_KR76_27125_WP_038682825.1 MPVTGADYKW MNLVARKPGK GLPRVLRRAA QGIGGMVIGR DYLAGGQALA AGLFDGALR- 218
KstD3_KR76_01140_WP_082003491.1 VVVLQSDYRW LST-GRRHWR GPLHMARIGL RFAWARLRRR RLVGLGSALV ASLMVGVRE- 233
KstD3_KR76_14500_WP_038679242.1 LIVTQADFRK ISL-GLRTLR GPLTMVKVML KRLLAIVTGK KMFAMGNAIA IGLRKGLAD- 219
kstD_Mtub_NP_218054.1 VVVMQQDYVR LNQLKRHP-R GVLRSMKVGA RTMWAKATGK NLVGMGRALI GPLRIGLQR- 218
Consensus VVVTQADYRW LXL-GRRPLR GPLRMDRVGX X--WA-VTGR DLXXGGXALX AXLRXGLAR-
Conservation 100% 0%
260 280 300
KstD1_KR76_02655_WP_038676428.1 EGGTIATECS VTGF----GR DAGGRLTTVR YDGPQGPAEV RARRGVLVAA GGFEGSPERR 240
KstD1_KR76_27115_WP_038682818.1 PHATLVRETA LAELVVEDGV VVGAIVE--- TDGV--RRAI RARRGVLLAA GGFEANDELR 242
KstD2_KR76_27125_WP_038682825.1 AGIPIWRETT LVELVTEGDR VVGAVLE--- RDGG--RVTV TARRGVVLAA GGFDHDMDMR 273
KstD3_KR76_01140_WP_082003491.1 AGIPLRLGTA MAGLVEDDGR VVGV------ RTGGGDR--V LARRGVVLAC GGFDHDAELR 285
KstD3_KR76_14500_WP_038679242.1 AGVPVEYDTA LLDLLVEDGR VVGVVVSTSS TDGGTERREI RARRGVILGS GGFEHSQELR 279
kstD_Mtub_NP_218054.1 AGVPVELNTA FTDLFVENGV VSGVYVRDSH EAESAEPQLI RARRGVILAC GGFEHNEQMR 278
Consensus AGXPXEXETA LXXLVVEDGR VVGVVVE--- XDGG-ERREX RARRGVXLAA GGFEHXXELR
Conservation 100% 0%
320 340 360
KstD1_KR76_02655_WP_038676428.1 AAHG-TPGRT EWSMAPRGTN TGEVIDAAAA AGAATDWSGE GWFCPGL-LQ PDGSGAFTLG 298
KstD1_KR76_27115_WP_038682818.1 QKYG-VPGVA RDTMGPPT-N VGAAHQAAIA VGADTDLMGE AWWSPGL-TH PDGRSAFALW 299
KstD2_KR76_27125_WP_038682825.1 HRYQ-AEFLD NWSLGNEG-N TGDAIKLAAE VGAELTLMDQ TWWFPAVAPL PGGTPQVLLA 331
KstD3_KR76_01140_WP_082003491.1 RTHQRRPAGP ELSLGVAT-N TGDGIRAAQV AGAATDLMDD AWWAPSI-PL PKG-PWFALS 342
KstD3_KR76_14500_WP_038679242.1 EKFLPQPTAA EWSTGAPA-N TGAGLLAGTA AGAATDLLDD AWWGPTI-PL P-GRAWFCLA 336
kstD_Mtub_NP_218054.1 IKYQRAPITT EWTVGASA-N TGDGILAAEK LGAALDLMDD AWWGPTVPLV --GKPWFALS 335
Consensus XKYQ-APGXX EWSXGXPX-N TGDGILAAAA AGAATDLMDD AWWGPXX-PL PDGRPWFALX
Conservation 100% 0%
380 400 420
KstD1_KR76_02655_WP_038676428.1 ---FRGGLIV DQSGQRYGNE CLPYDRFGRV MA-------Q APE----RTP S-WFVFDSRE 343
KstD1_KR76_27115_WP_038682818.1 ---FTGGIFV DGAGRRFVNE SAPYDRLGRA VI----DHLT EGG----VTP RYWMVYDHK- 347
KstD2_KR76_27125_WP_038682825.1 ERSLPGSIMV DGHGRRFINE STDYMTFGQT VL----GRDR AGD----PVG SMWLVFDQSY 383
KstD3_KR76_01140_WP_082003491.1 ERSLPGGIIV NRHGERFMNE SLPYVEATHR MFGGTHGRGT GPDDDAENLP C-WLVFDQTY 401
KstD3_KR76_14500_WP_038679242.1 ERNLPGSIIV NSAGQRYMNE ALPYVEATHE IY---KGQAT GVD----HVP S-YMVFDTHY 388
kstD_Mtub_NP_218054.1 ERNSPGSIIV NMSGKRFMNE SMPYVEACHH MYGGEHGQGP GPG---ENIP A-WLVFDQRY 391
Consensus ERXLPGXIIV XGXGXRFMNE SLPYVEAGHX MY----GXGT GPD----NXP S-WLVFDQXY
Conservation 100% 0%
440 460 480
KstD1_KR76_02655_WP_038676428.1 EGRLPAIAMP EGERA---EH LAAGTWVEAE TLDELAARAG LDPAALTATV ARFNELAANG 400
KstD1_KR76_27115_WP_038682818.1 EGSIPPVRAT NVSMVDEEQY VAAGLWHTAD TLPELAALIG VPADALVATV ARFNELVADG 407
KstD2_KR76_27125_WP_038682825.1 RNSYVLAGSL FPRMALPQEW YDAGIAHRAG TAAELARAAG LPEDAFTATL RRFNTMAAAG 443
KstD3_KR76_01140_WP_082003491.1 RDRYLFAGV- QPGRALPKSW LASGAITKAG SLDELAAAIG VPAAGLAATV ARFNGFAAEG 460
KstD3_KR76_14500_WP_038679242.1 RNRYLFAGV- AGRQPFPGRW YKEGVVVRAD TLAELAAKVG LPEGSLDATV DRFNGFARSG 447
kstD_Mtub_NP_218054.1 RDRYIFAGL- QPGQRIPSRW LDSGVIVQAD TLAELAGKAG LPADELTATV QRFNAFARSG 450
Consensus RXRYXFAGV- QPXXALPXXW LAAGVXVRAD TLAELAAXAG LPADALTATV ARFNXFAASG
Conservation 100% 0%
500 520 540
KstD1_KR76_02655_WP_038676428.1 KDEDFGRGED EYDTFFAS-A GSPNKALVPL DRAPYTAARI VLSDLGTKGG LVTDASARVL 459
KstD1_KR76_27115_WP_038682818.1 YDADFGRGGE AYDRFFSG-G EPP---LVSI DEGPFHAAAF GISDLGTKGG LRTDTSARVL 463
KstD2_KR76_27125_WP_038682825.1 IDDDFHRGNS AYDRYYGDPT VTPNPNLRPL DRGDLYAVKV VLSDLGTCGG LSADELGRPL 503
KstD3_KR76_01140_WP_082003491.1 VDHDHHRGES AYDHYYGDLT NKPNPSLGPI AKGPFYAARM VPADLGTKGG IRTDPRARAL 520
KstD3_KR76_14500_WP_038679242.1 TDEDFHRGES AYDKYYSDPT VKPNCSLAPI EKGPFYAVKI VPGDLGTKGG LVTDERARVL 507
kstD_Mtub_NP_218054.1 VDEDYHRGES AYDRYYGDPS NKPNPNLGEV GHPPYYGAKM VPGDLGTKGG IRTDVNGRAL 510
Consensus VDEDFHRGES AYDRYYGDPT XKPNPXLXPI DXGPFYAAKX VPSDLGTKGG LRTDEXARVL
Conservation 100% 0%
560 580
KstD1_KR76_02655_WP_038676428.1 DGDGVPIPGL YAASNSTASV FGSAYPGPGA PLGAAMTFAS LAVEDLLG-- ------ 507
KstD1_KR76_27115_WP_038682818.1 TADGTPIGGL YAAGNTMAAP SGTTYPGGGN PIGTSMLFSH LAVRHMGTED AR---- 515
KstD2_KR76_27125_WP_038682825.1 RADGSPIEGL YAIGNTAGNV FGRSYPGAGA TIGQGLVFGH IVATHAATA- ------ 552
KstD3_KR76_01140_WP_082003491.1 RADGTPIPGL YAAGNTSAAV MGHTYAGPGA TIGPALVFGY LAALDCAAVR QDEAHD 576
KstD3_KR76_14500_WP_038679242.1 REDGSVIPGL YAAGNVSAAV MGNTYPGPGG TIGPAIVFGY LAAEDLTKER S----- 558
kstD_Mtub_NP_218054.1 RDDGSIIDGL YAAGNVSAPV MGHTYPGPGG TIGPAMTFGY LAALHIADQ- --AGKR 563
Consensus RADGSPIPGL YAAGNTSAAV MGHTYPGPGA TIGPAMVFGY LAAXXLATE- ------
Conservation 100% 0%

Supplementary Figure S3. KstD alignment

The important residues of KstD2 from *R. erythropolis* SQ1 [86] are the following: FAD-binding domain (12-35 in product of Rv3537 of *M. tuberculosis*), consensus GSG(A/G)(A/G)(A/G)X_{17}E; Ser325 (318A in product of Rv3537 of *M. tuberculosis* or T in other actinomycetal KSTD enzymes, i.e., small residue at this position); and Thr503 (497 in product of Rv3537 of *M. tuberculosis*). *KR76_27125* of *N. simplex* contains Ala in position corresponding to 318A in *M. tuberculosis* and 325S in *R. erythropolis*; but FAD-binding domain is different from consensus: GSGT-GX_{17}E.
